# Longitudinal *in vivo* (*R*)-[^18^F]FBFP PET imaging for preclinical evaluation of cerebral sigma-1 receptor after ischemic stroke

**DOI:** 10.7150/thno.117418

**Published:** 2026-01-01

**Authors:** Jingfei Yang, Luoxia Liu, Huimin Zhou, Chuanzhi Huang, Dongdong Wang, Ziqiang Wang, Yifan Shi, Shuang Song, Xiaoyun Deng, Yuankai Zhu, Jun Zhao, Zhouping Tang, Hongmei Jia, Xiaohua Zhu

**Affiliations:** 1 Department of Nuclear Medicine, Tongji Hospital, Tongji Medical College, Huazhong University of Science and Technology. Wuhan 430030, China.; 2 National Medical Center for Major Public Health Events. Wuhan 430030, China.; 3 Department of Neurology, Tongji Hospital, Tongji Medical College, Huazhong University of Science and Technology. Wuhan 430030, China.; 4 Key Laboratory of Radiopharmaceuticals (Beijing Normal University), Ministry of Education, College of Chemistry, Beijing Normal University. Beijing 100875, China.; 5 State Key Laboratory for Diagnosis and Treatment of Severe Zoonotic Infectious Disease. Wuhan 430030, China.

**Keywords:** ischemic stroke, sigma 1 receptor, (*R*)-[^18^F]FBFP, positron emission tomography, neurological outcome

## Abstract

**Rationale:** Sigma-1 receptor (sigma-1R) is a promising biomarker and therapeutic target for ischemic stroke. However, the real-time changes in the expression of sigma-1R post-stroke have not been elucidated. (*R*)-1-(4-[^18^F]Fluorobenzyl)-4-[(tetrahydrofuran-2-yl)methyl]piperazine ((*R*)-[^18^F]FBFP) has emerged as a novel radiotracer targeting sigma-1R. This study aimed to use (*R*)-[^18^F]FBFP PET imaging for the investigation of spatiotemporal alterations in sigma-1R expression in the rat brain following stroke and treatment, and to explore the correlation between the imaging findings and neurological outcomes.

**Methods:** Sigma-1R levels were evaluated on days 1, 3, 7, 14, 21, and 28 after middle cerebral artery occlusion (MCAO) using (*R*)-[^18^F]FBFP PET/CT imaging. *Ex vivo* autoradiography and immunofluorescence (IF) staining were performed to corroborate the findings from PET/CT imaging. The cellular localization of sigma-1R during stroke progression was identified by co-labeling sigma-1R with neurons (NeuN), astrocytes (GFAP), and microglia (Iba1). Behavioral tests were conducted on MCAO rats at corresponding time points, and the correlation between PET signals and neurological outcomes was analyzed. The MCAO rats were then treated with recombinant tissue-type plasminogen activator (rtPA), and the therapeutic response was evaluated with (*R*)-[^18^F]FBFP to elucidate the impact of treatment on PET imaging.

**Results:** Compared with the sham group, the ipsilateral-to-contralateral hemisphere uptake ratio of (*R*)-[^18^F]FBFP of the MCAO group significantly decreased in the acute phase (days 1 and 3), increased in the subacute phase (days 7 and 14), and then gradually declined in the chronic phase (days 21 and 28). The PET imaging findings were in agreement with the *ex vivo* autoradiography and IF staining. Changes in sigma-1R levels in ischemic lesions were influenced by the initial neuronal loss and the later accumulation of glial cells. Furthermore, there was a significant correlation between the uptake of (*R*)-[^18^F]FBFP and the neurological outcomes during stroke recovery. After rtPA treatment, the (*R*)-[^18^F]FBFP uptake in the affected hemisphere gradually returned to levels comparable to the contralateral hemisphere.

**Conclusions:** (*R*)-[^18^F]FBFP PET imaging effectively visualized and accurately quantified the spatiotemporal alterations of sigma-1R in the rat brain during ischemic stroke progression. The (*R*)-[^18^F]FBFP uptake correlated with the neurological outcomes during stroke recovery, and (*R*)-[^18^F]FBFP PET imaging could be a valuable tool for predicting post-stroke recovery and evaluating the efficacy of rtPA treatment.

## Introduction

Ischemic stroke, caused by cerebrovascular occlusion from embolism or thrombosis, results in reduced blood flow to local brain tissue and severe neurological deficits 1-3. As one of the most common and devastating acute cerebrovascular diseases, ischemic stroke presents high morbidity, mortality, and disability rates, imposing a substantial burden on healthcare systems worldwide 2. Vascular recanalization treatments, including mechanical thrombectomy and intravenous thrombolytic therapy with recombinant tissue plasminogen activator (rtPA), can improve neurological outcomes 1. However, only a small proportion of patients eventually achieve full recovery of daily activities, and rehabilitation remains a challenge 2. The limited regenerative capacity of brain tissue and scarcity of treatment options highlight the urgent need for novel therapeutic targets and strategies to improve stroke outcomes.

Sigma-1 receptor (sigma-1R) is a molecular chaperone protein predominantly located on the mitochondria-associated endoplasmic reticulum membrane (MAM). It regulates Ca^2+^ signaling and lipid exchange between the endoplasmic reticulum (ER) and mitochondria, and therefore is essential for cellular homeostasis 4, 5. Sigma-1R is involved in a variety of cellular processes, including neurotransmission, ER stress, autophagy, inflammation, mitochondrial dysfunction, oxidative stress, and apoptosis 4-6. It is abundantly present in the central nervous system (CNS) and plays a vital role in regulating diverse neurological disorders, in particular ischemic stroke 6-8. Preclinical studies have shown that sigma-1R activation exerts neuroprotective effects in ischemic stroke by multiple mechanisms, such as promoting neurite growth, mitigating ER stress-induced apoptosis, supporting astrocyte survival and oligodendrocyte generation, and modulating neuroinflammation 9-13. A Phase II trial indicated that cutamesine (SA4503), a sigma-1R agonist, improved functional outcomes in patients with severe stroke 14. The spatiotemporal alterations in sigma-1R expression may affect stroke progression and neurofunctional recovery, underscoring the need for longitudinal *in vivo* assessment of sigma-1R levels. Conventional methods for sigma-1R detection could not non-invasively and repeatedly monitor spatiotemporal evolution within the same subject, hampering the comprehensive evaluation. Therefore, innovative detection strategies are urgently warranted to capture real-time changes in sigma-1R after stroke.

Positron emission tomography (PET) offers a powerful non-invasive approach for *in vivo* imaging, presenting high sensitivity, accurate quantification, and real-time monitoring capabilities. PET imaging targeting sigma-1R enables longitudinal tracking its distribution and expression in the brain of the same subject following stroke, aiding in stroke pathophysiology research. Several ^11^C- and ^18^F-labeled sigma-1R radiotracers have been developed, including [^11^C]SA4503, [^11^C]PB212, [^11^C]HCC0923, [^11^C]HCC0929, [^18^F]FM-SA4503, [^18^F]FPS, [^18^F]FTC-146, [^18^F]Fluspidine, [^18^F]IAM6067, and [^18^F]CNY-05 15-17. Only a small fraction of these radiotracers, however, have progressed to clinical stages, and their application is constrained by slow clearance from the brain and remains unexplored in the context of stroke 15-18. The development of new PET radiotracers targeting cerebral sigma-1R is therefore an unmet medical need. In the previous study, both enantiomers of 1-(4-[^18^F]Fluorobenzyl)-4-[(tetrahydrofuran-2-yl)methyl]piperazine ([^18^F]FBFP), (*S*)- and (*R*)-[^18^F]FBFP, exhibit high affinity and specificity for sigma-1R, making them feasible tools for the non-invasive quantification of sigma-1R in the brain 19. (*R*)-[^18^F]FBFP, in particular, demonstrates a high brain uptake, favorable brain-to-blood ratios, and metabolic stability in the brain 19. However, its application in ischemic stroke has not yet been reported.

Consequently, we aim to use (*R*)-[^18^F]FBFP to track the spatiotemporal variations of sigma-1R after ischemic stroke, to examine its correlation with neurological outcomes, and to evaluate its potential as an imaging marker for stroke progression. We established a rat middle cerebral artery occlusion (MCAO) model and conducted dynamic PET/computed tomography (CT) imaging to determine the optimal imaging time points after (*R*)-[^18^F]FBFP administration. MCAO rats were then subjected to longitudinal (*R*)-[^18^F]FBFP and [^18^F]FDG PET/CT imaging, neurobehavioral tests, immunofluorescence (IF) staining, *ex vivo* autoradiography, and Nissl staining. Additionally, we investigated the effect of intravenous rtPA treatment on (*R*)-[^18^F]FBFP brain uptake after stroke to explore the potential of this novel radiotracer for monitoring therapeutic efficacy. Our study revealed the molecular alterations related to stroke pathophysiology, which could advance the development of diagnostic tools and therapeutic interventions involving sigma-1R in stroke.

## Methods

### Radiolabeling and quality control

**Figure 1:**
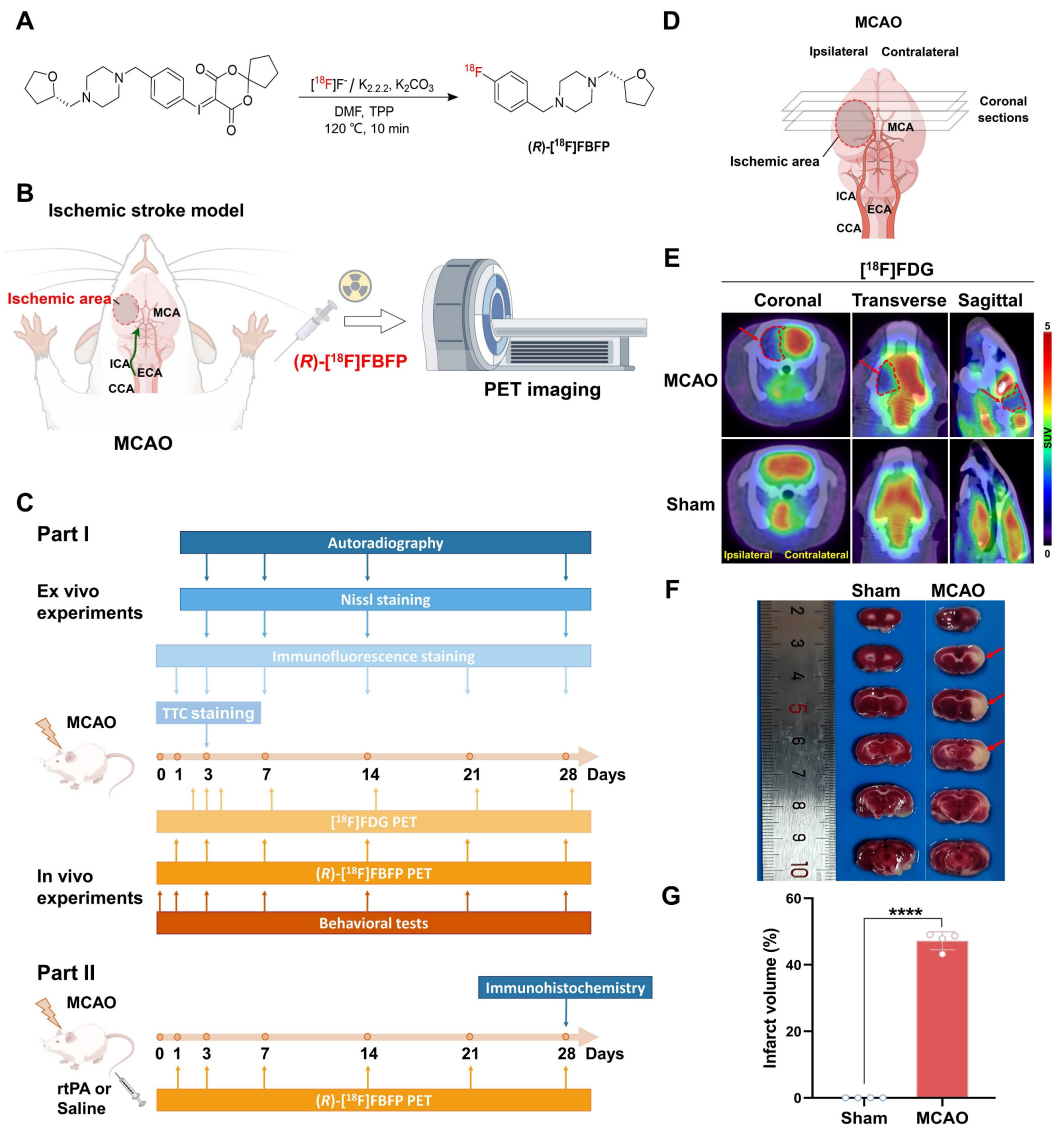
** Experimental outline schematic and validation of MCAO rats.** (A) Radiosynthesis of (*R*)-[^18^F]FBFP. (B) Diagram of the MCAO model and PET imaging in rats. (C) Overview of the experimental process. (D) Schematic of coronal brain sections post-MCAO. (E) [^18^F]FDG PET/CT images of brains in rats on day 3 post-stroke in MCAO and sham groups, with ischemic regions highlighted by red arrows. (F) TTC-stained brain sections from MCAO and sham groups on day 3, with infarcted areas as unstained. (G) Quantification of infarct volume (%) by TTC staining. Schematic diagrams were created by Figdraw. Values are mean ± SD (n = 4/group). ****P < 0.0001.

The radiosynthesis of (*R*)-[^18^F]FBFP (Figure 1A) was performed as previously described 19. [^18^F]Fluoride was first collected on a QMA cartridge (Waters Corporation, USA), then eluted with a solution of Kryptofix 2.2.2/K_2_CO_3_ (1 mL, containing 13 mg of Kryptofix 2.2.2 and 1.1 mg of K_2_CO_3_ in a 4:1 (v/v) CH_3_CN/H_2_O solvent). The solvent was removed at 120 °C for 20 min using a stream of nitrogen gas and then added with anhydrous acetonitrile (3 × 1 mL, 120 °C) in a reaction vessel. Then, 2.0 mg of precursor, 3.75 mg of triphenylphosphine (PPh_3_, TPP), and 0.5 mL of anhydrous N, N-dimethylformamide (DMF) were added to the vessel, and the mixture was heated at 120 °C for 10 min. Afterward, the reaction was diluted with 1.8 mL of CH_3_CN and 1.2 mL of H_2_O, and the resulting solution was injected into a high-performance liquid chromatography (HPLC) system. The product was purified by a Waters XBridge BEH C18 OBD Prep column (5 μm, 10 mm × 250 mm) using a mobile phase consisting of 60% water and 40% acetonitrile and containing 0.1% triethylamine at a flow rate of 4 mL/min. Fractions containing the ^18^F-labeled product were collected and diluted with 20 mL of H_2_O, then adsorbed on an activated Sep-Pak C18 cartridge (Waters Corporation, USA). The product was subsequently eluted with 1 mL of ethanol and dried under a nitrogen stream. Finally, the product was reconstituted in sterile saline containing approximately 7% ethanol for further experiments. All the reagents and chemicals were commercially sourced without additional purification. Radiochemical purity (RCP) and stability of (*R*)-[^18^F]FBFP were measured using analytical HPLC, and detailed experimental procedures were provided in the Supplemental Data.

### Animals

The experimental process is illustrated in Figure 1B, C. In Part I, adult male Sprague-Dawley rats (270 ± 20 g) were randomized into MCAO and sham groups. In Part II, MCAO rats were randomized into two groups: MCAO + Vehicle, with saline administration, and MCAO + rtPA, where rtPA (3 mg/kg, 1 mg/mL, Alteplase, Boehringer-Ingelheim, Germany) was intravenously injected at 4 h post-occlusion 20. Rats were housed under standard conditions (12-hour light/dark cycle) with free access to food and water. All experiments were carried out in compliance with the Institutional Animal Care and Use Committee (Tongji Hospital, Tongji Medical College, Huazhong University of Science and Technology, China; TJH-202304016) and the ARRIVE guidelines 2.0 (Animal Research: Reporting of In Vivo Experiments).

### MCAO model

Focal cerebral ischemia was induced by MCAO as previously reported 21. Male rats were anesthetized using isoflurane/O_2_ (5% induction, 1% maintenance) and placed on a heating pad throughout the MCAO surgery. The anterior neck was incised along the median line with a 2-3 cm cut, and the right unilateral MCAO was achieved by inserting a monofilament (Guangzhou Jialing Biotechnology Co., Ltd., China) into the internal carotid artery (ICA) through the external carotid artery (ECA) to block the middle cerebral artery (MCA). After 90 min, the monofilament was withdrawn to allow reperfusion, and the wound was stitched up using 4-0 sutures. Postoperatively, rats were kept under a heat lamp until fully awake. Topical erythromycin ointment was applied to the surgical site to prevent infection. Adequate measures were taken to minimize pain or discomfort during surgeries, and the rats were supplied with *ad lib* food and water during recovery. The Zea-Longa scoring system assigned scores as follows: 0 for no obvious neurological deficits; 1 denoted an inability to fully extend the left forelimb; 2 for walking in circles; 3 for walking imbalance; 4 for limb paralysis, inability to walk, or loss of consciousness; and 5 for death. For inclusion in our study, animals were required to have scores between 1 and 4, signifying successful model induction. Consequently, scores of 0 and 5 were established as exclusion criteria. The surgical failure rate was approximately 10-20%. In the sham group, rats experienced identical surgical procedures without a monofilament to block blood flow in the MCA. Researchers were blinded to the experimental groups during the analysis.

### TTC staining

TTC staining was employed to assess infarct size in coronal brain sections (Figure 1D) 21. ImageJ software (National Institutes of Health, Bethesda, Maryland) was used to quantify the infarct size as a percentage of the ipsilateral hemisphere.

### PET/CT imaging

PET/CT imaging was performed using the u-EXPLORE PET/CT system (Shanghai United Imaging Healthcare Co., Ltd., China). Male rats were anesthetized with isoflurane/O_2_ at a flow rate of 1-2 L/min and maintained in the prone position on the scanning bed throughout the scanning process. Immediately after intravenous injection of (*R*)-[^18^F]FBFP (11.1-14.8 MBq, formulated in 0.6 mL saline with 7% ethanol), dynamic PET/CT imaging was performed. The dynamic PET scans for (*R*)-[^18^F]FBFP lasted for 90 min, during which the breathing rate and body temperature of rats were monitored, and the isoflurane dose was modified as needed. Dynamic PET/CT imaging was used to determine the time window when the radiotracer remained metabolically stable in the brain. Time-activity curves (TACs) were derived from the full dynamic dataset (n = 4~5/group). Moreover, 30 min post-injection of (*R*)-[^18^F]FBFP, rats were anesthetized and placed on the scanning bed for a 10-minute PET scan (n = 7~10/group). The images were reconstructed with higher resolution using the ordered subsets expectation maximization (OSEM) algorithm and analyzed with the u-EXPLORE workstation. The region of interest (ROI) in the brain was manually outlined, and its standardized uptake value (SUV) was measured. PET imaging data were presented as SUV_mean_ and SUV_max_ to assess (*R*)-[^18^F]FBFP uptake levels. In addition, *in vivo* [^18^F]FDG PET/CT imaging was performed after stroke. All rats were fasted overnight for at least 12 h before [^18^F]FDG PET/CT scans. The rats were anesthetized and placed on the scanning bed 60 min after the injection of [^18^F]FDG, and PET images were acquired for 10 min.

The blocking studies with the sigma-1R selective agonist SA4503 (MedChemExpress, USA) were conducted to determine the *in vivo* binding specificity of (*R*)-[^18^F]FBFP. SA4503 (5 μmol/kg) was co-injected with (*R*)-[^18^F]FBFP (11.1-14.8 MBq, formulated in 0.6 mL saline containing 7% ethanol) into rats of the sham and MCAO groups on days 7 or 14 post-stroke (n = 4~6/group). Thirty minutes after the co-injection, the rats were anesthetized and placed on the scanning bed for (*R*)-[^18^F]FBFP PET/CT imaging.

### Behavioral tests

Neurobehavioral tests were carried out by the same investigator, blind to the group allocation at baseline and on days 1, 3, 7, 14, 21, and 28 after MCAO. The modified neurological severity score (mNSS) was used to evaluate motor abilities, sensory disturbance, reflexes, and balance 22. The adhesive removal test was performed to assess forepaw sensitivity and motor deficits 23. The cylinder test was used to measure forepaw asymmetry 22. The corner test was conducted to assess sensorimotor function, which integrated both stimulations of the vibrissae (sensory/neglect) and rearing (motor response) 24. Detailed experimental procedures were provided in the Supplemental Data.

### Immunofluorescence staining

Rats were euthanized by transcardial perfusion with phosphate-buffered saline (PBS) followed by 4% paraformaldehyde (PFA) (Servicebio, China) (n = 5~6/group). Brains were post-fixed in 4% PFA overnight, embedded in paraffin, and sectioned coronally at 5 µm. After deparaffinization and rehydration, antigen retrieval was performed in high-temperature and high-pressure ethylene diamine tetraacetic acid (EDTA) solution (pH 9.0; Beyotime, China). Sections were permeabilized and blocked with 10% serum, then incubated overnight at 4 °C with primary antibodies: anti-Sigma-1R (1:100, Cat#sc-137075, Santa Cruz Biotechnology, USA), anti-NeuN (1:500, Cat#ab177487, Abcam, UK), anti-GFAP (1:500, Cat#ab68428, Abcam, UK), anti-Iba1 (1:500, Cat#ab178846, Abcam, UK), anti-TSPO (1:100, Cat#abs124730, absin, China), anti-iNOS (1:100, Cat#ab283655, Abcam, UK), and anti-CD206 (1:100, Cat#24595, CST, USA). Following this, the sections were washed three times with TBST for 5 min each. After washing, sections were incubated with appropriate secondary antibodies at 37 °C for 45 min, counterstained with 4,6-diamidino-2-phenylindole (DAPI; Solarbio, China), and mounted. Finally, the stained sections were imaged using a fluorescence microscope (Olympus BX53, Tokyo, Japan). Images were analyzed using ImageJ software. ROIs included the infarct area, peri-infarct regions, and contralateral homologous areas as internal controls. The infarct area was delineated approximately by tissue loss, structural deformation, and NeuN-negative area. Due to extensive neuronal loss in the infarct area, analysis of sigma-1R localization in neurons was focused on the peri-infarct regions, where surviving neurons allow reliable IF assessment. Within each ROI, representative fields were chosen to ensure reliable quantification. The same criteria were applied consistently across all experimental animals.

### *Ex vivo* autoradiography

Autoradiography 25 was performed on days 3, 7, 14, and 28 after MCAO surgery. The 20-µm-thick coronal brain sections were incubated in Tris-HCl buffer (50 mmol/L, pH 7.4) at room temperature for 10 min and dried at 37 °C. After the addition of (*R*)-[^18^F]FBFP (3.7 MBq in 1 mL of buffer), the sections were incubated at room temperature for 60 min. Then, the sections were washed three times with distilled water for 5 min each and dried at 37 °C. Subsequently, the processed sections were placed on a phosphor screen (Amersham Typhoon, Cytiva, USA) and exposed for 120 min. Finally, the autoradiograms were scanned, and quantification was performed using image analysis software (ImageJ, National Institutes of Health, Bethesda, Maryland).

### Gene expression profiling of *Sigmar1*

Gene expression data of *Sigmar1* in rats and mice with ischemic stroke were obtained from the Gene Expression Omnibus (GEO) database (http://www.ncbi.nlm.nih.gov) and further analyzed (datasets GSE36010 26, GSE196737 27, and GSE35338 28).

### Statistical analysis

Results are presented as mean ± standard deviation (SD). Statistical analyses were performed using Student's t-test (two-tailed) and one-way analysis of variance (ANOVA) with Tukey's post hoc test, all conducted with GraphPad Prism 8 (GraphPad Software Inc., USA). Normality was tested using Shapiro-Wilk test, and non-normal data were analyzed by non-parametric tests. *P* < 0.05 was considered statistically significant.

## Results

### Radiosynthesis and quality control of (*R*)-[^18^F]FBFP

The radiosynthesis of (*R*)-[^18^F]FBFP was performed as shown in Figure 1A. The decay-corrected radiochemical yield (RCY) was 20.1% ± 5.1% (n = 6), with the RCP > 98% by radio-HPLC (Figure S1A). Molar activity ranged from 99 to 191 GBq/μmol. *In vitro* stability of (*R*)-[^18^F]FBFP in serum or PBS was confirmed by HPLC, with chromatograms showing a single peak (Figure S1B).

### Dynamic *in vivo* PET/CT imaging of MCAO rats

On day 3 post-stroke, [^18^F]FDG PET/CT imaging revealed a reduced glucose metabolism in the infarct area of MCAO rats, in contrast to the normal uptake of [^18^F]FDG in the sham group (Figure 1E). TTC staining confirmed a large infarct lesion in the MCAO group. The results confirmed a successful stroke induction in both cortical and subcortical areas (Figure 1F-G).

**Figure 2:**
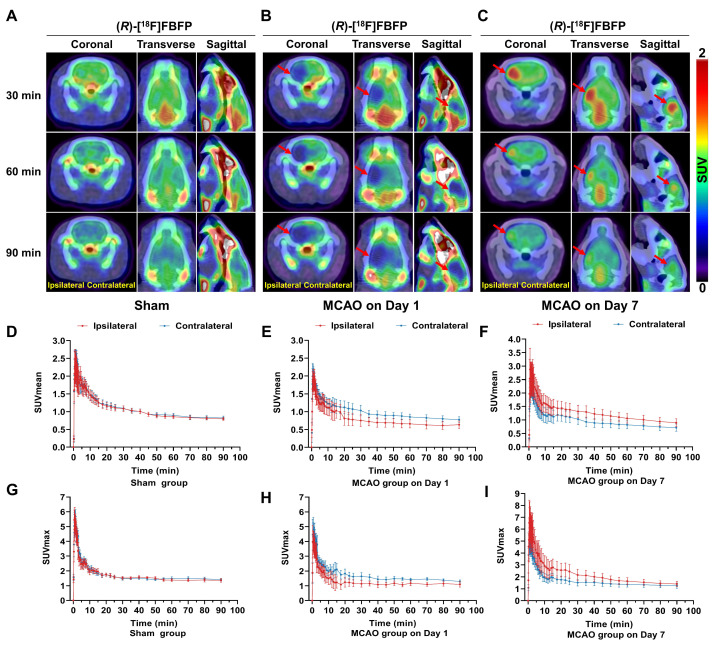
** Dynamic PET/CT imaging of MCAO rats.** (*R*)-[^18^F]FBFP PET/CT images of brains from the sham (A) and MCAO groups on days 1 (B) and 7 (C) post-stroke at 30, 60, and 90 min after injection. Red arrows indicate the ischemic lesions. TACs of (*R*)-[^18^F]FBFP in ipsilateral and contralateral hemispheres for the sham group (D, G) and MCAO group on days 1 (E, H) and 7 post-stroke (F, I). Values are mean ± SD (n = 4~5/group). MCAO, middle cerebral artery occlusion; SUV, standardized uptake value; TACs, time-activity curves.

Dynamic PET/CT imaging of (*R*)-[^18^F]FBFP was performed over 90 min after tracer injection, and the TACs of (*R*)-[^18^F]FBFP were shown in Figure 2. In the sham group, the (*R*)-[^18^F]FBFP uptake peaked within 2 min and gradually decreased over time, with similar SUV_mean_ and SUV_max_ in both hemispheres (Figure 2A, D, G). In the MCAO group on day 1 post-stroke, the (*R*)-[^18^F]FBFP uptake in the ipsilateral hemisphere was lower than that in the contralateral one (Figure 2B, E, H). Conversely, the (*R*)-[^18^F]FBFP uptake in the ipsilateral hemisphere was significantly higher than that in the contralateral hemisphere on day 7 post-stroke (Figure 2C, F, I). A consistently high cerebral signal was noted between 30 and 40 min post-injection. This time window was then selected for later static acquisitions.

### MCAO rats exhibited a time-related progression in brain uptake of (*R*)-[^18^F]FBFP

**Figure 3:**
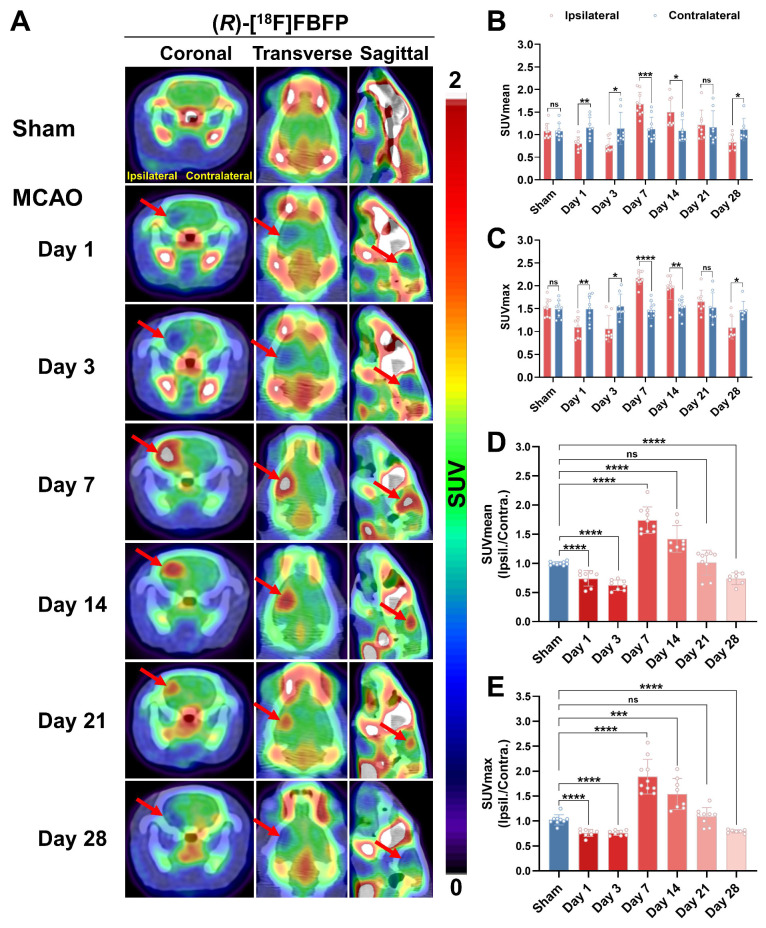
** Spatiotemporal variations in (*R*)-[^18^F]FBFP uptake in the brain after stroke.** (A) (*R*)-[^18^F]FBFP PET/CT images of brains from the sham and MCAO groups on days 1, 3, 7, 14, 21, and 28 post-stroke, with red arrows highlighting ischemic lesions in the ipsilateral hemispheres. (B, C) Quantification of (*R*)-[^18^F]FBFP brain uptake (SUV_mean_ in B, SUV_max_ in C). (D, E) Ratios of (*R*)-[^18^F]FBFP uptake (SUV_mean_ in D, SUV_max_ in E) in the ipsilateral hemisphere relative to the contralateral hemisphere. Values are mean ± SD (n = 7~10/group). *P < 0.05, **P < 0.01, ***P < 0.001, ****P < 0.0001, and ns: no significance.

Longitudinal PET/CT imaging revealed a dynamic change of (*R*)-[^18^F]FBFP uptake in ischemic lesions. The tracer uptake in the ipsilateral side was lower than that of the contralateral side on days 1 and 3 post-stroke. It surpassed the latter starting from day 7, remained at a similar level until day 21, and then dropped once again below the latter on day 28 (Figure 3A-C). A similar trend was observed in the ipsilateral-to-contralateral uptake ratio after stroke. Compared to the sham group (1.00 ± 0.03), the ipsilateral-to-contralateral uptake ratio (SUV_mean_ in Figure 3D, n = 7~10) in the MCAO group was lower on days 1 (0.74 ± 0.13, *P* < 0.0001) and 3 (0.63 ± 0.09, *P* < 0.0001) post-stroke, yet higher on days 7 (1.74 ± 0.23, *P* < 0.0001) and 14 (1.42 ± 0.23, *P* < 0.0001). At later time points (days 21 and 28), this ratio in the MCAO group gradually decreased, with the ratio on day 28 (0.74 ± 0.10, *P* < 0.0001) being significantly lower than the sham group. The SUV_max_ ratio presented a similar trend (Figure 3E). The PET imaging findings were in line with the results of *ex vivo* autoradiography of (*R*)-[^18^F]FBFP. Compared with those of the sham group, the MCAO group showed higher radioactivity accumulation in rat brains on days 7 and 14 post-stroke, but lower signals on days 3 and 28 (Figure S2). These results suggested that (*R*)-[^18^F]FBFP uptake decreased in the acute phase, increased in the subacute phase, and gradually declined in the chronic phase.

**Figure 4:**
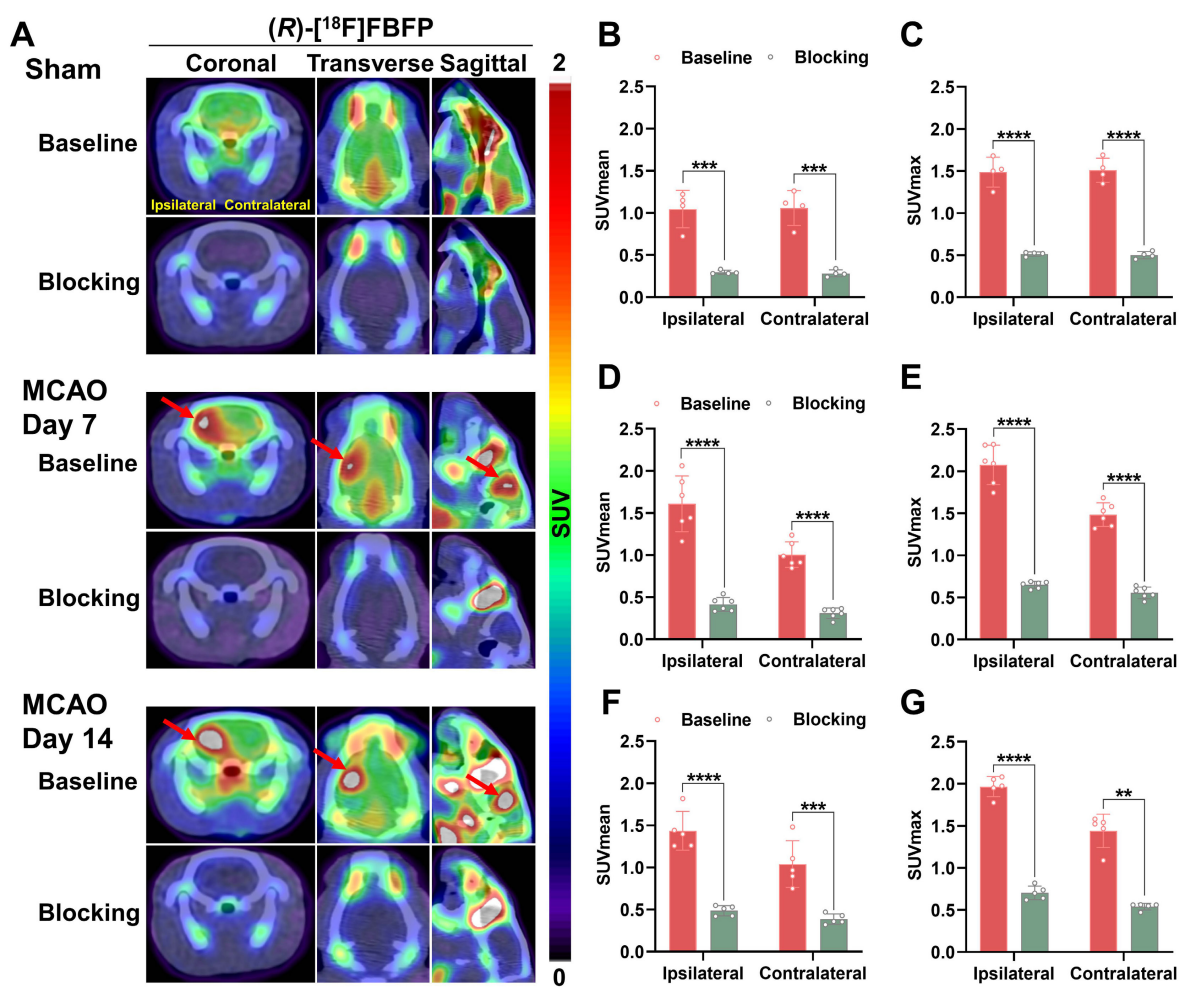
** Blocking studies of (*R*)-[^18^F]FBFP in MCAO rats.** (A) Representative coronal, transverse, and sagittal (*R*)-[^18^F]FBFP PET/CT images of rat brains under baseline or blocking conditions (with co-injection of SA4503 at 5 μmol/kg, iv) in the sham group and the MCAO group on days 7 or 14 after stroke. Red arrows indicate ischemic lesions in the ipsilateral hemisphere. (B-G) Quantification of (*R*)-[^18^F]FBFP brain uptake (SUV_mean_ in B, D, F, and SUV_max_ in C, E, G). Values are mean ± SD (n = 4~6/group). **P < 0.01, ***P < 0.001, and ****P < 0.0001. MCAO, middle cerebral artery occlusion; SUV, standardized uptake value.

To assess the *in vivo* binding specificity to sigma-1R, SA4503, an agonist for sigma-1R, was used to block sigma-1R 19. Co-injection of SA4503 with (*R*)-[^18^F]FBFP significantly reduced ipsilateral PET signals in both the sham (n = 4) and MCAO groups on days 7 (n = 6) and 14 (n = 5) post-stroke, confirming specific binding of (*R*)-[^18^F]FBFP to sigma-1R. A similar decrease in PET signals was observed in the contralateral hemisphere upon blocking (Figure 4).

### Sigma-1R levels in MCAO rats varied on different days after stroke

**Figure 5:**
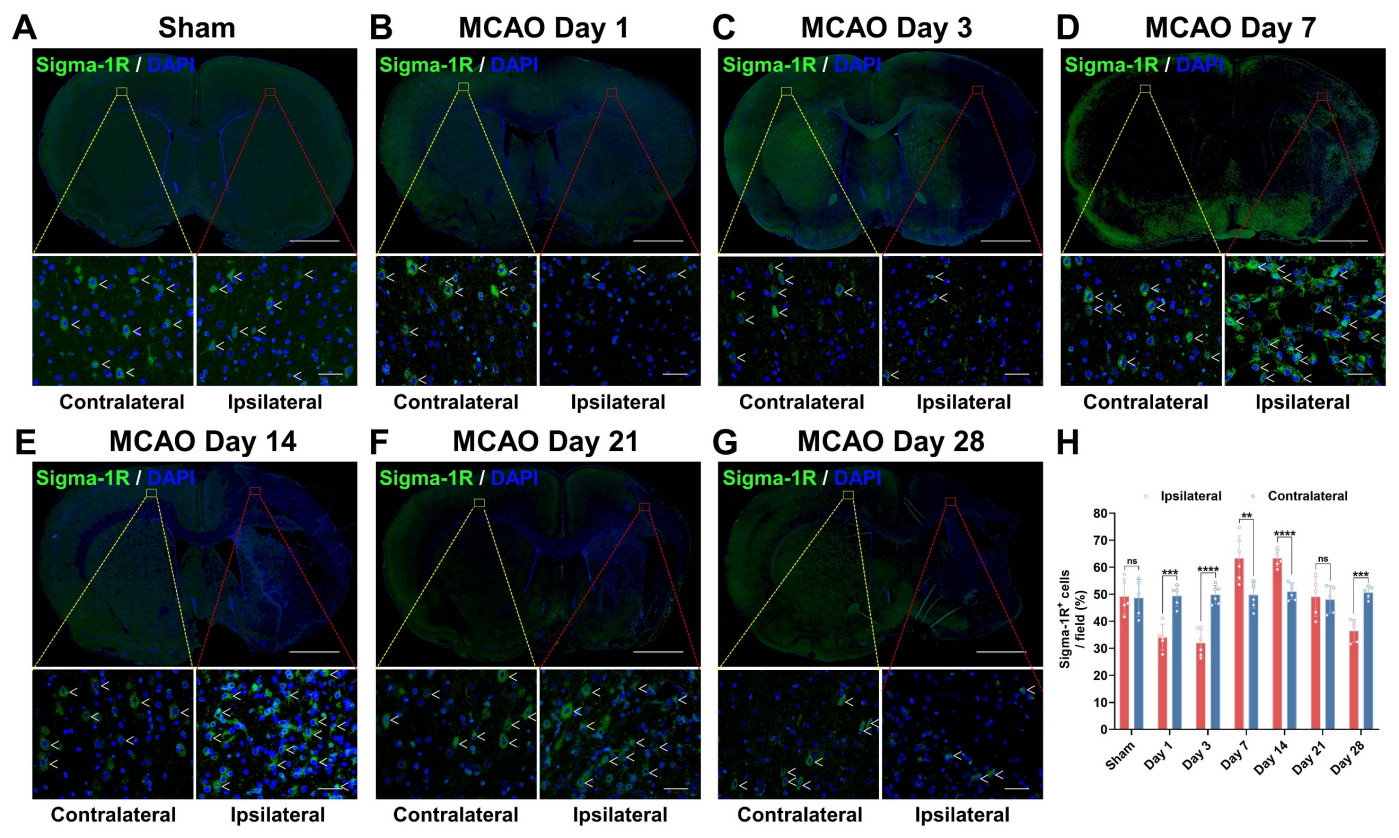
** Variations in sigma-1R expression after stroke.** (A-G) Immunofluorescence images of sigma-1R (green) in the ipsilateral versus contralateral hemispheres of rats from the sham (A) and MCAO groups on days 1 (B), 3 (C), 7 (D), 14 (E), 21 (F), and 28 (G) post-stroke. White arrows indicate sigma-1R^+^ cells. Scale bars, 2 mm or 50 μm. (H) Quantification of sigma-1R^+^ cells per field. Values are mean ± SD (n = 5~6/group). **P < 0.01, ***P < 0.001, ****P < 0.0001, and ns: no significance.

Sigma-1R levels were then assessed using IF staining (Figure 5). The sham group showed no notable difference in sigma-1R expression between both hemispheres (*P* = 0.8766, n = 5; Figure 5A). In the MCAO group (n = 6), sigma-1R expression in the ipsilateral ischemic lesions was lower than that in the contralateral tissue on days 1 (*P* = 0.0006) and 3 (*P* < 0.0001) post-stroke, became higher than the latter on days 7 (*P* = 0.0055) and 14 (*P* < 0.0001), and then declined again to a lower level on day 28 (*P* = 0.0002) (Figure 5). This dynamic change matched the results of PET imaging and autoradiography. Moreover, the GSE36010 dataset demonstrated reduced *Sigmar1* mRNA expression in the core infarct area of permanent MCAO on day 3, compared to the sham tissues (Figure S4A).

### Changes in the cellular localization of sigma-1R in the brain of MCAO rats

**Figure 6:**
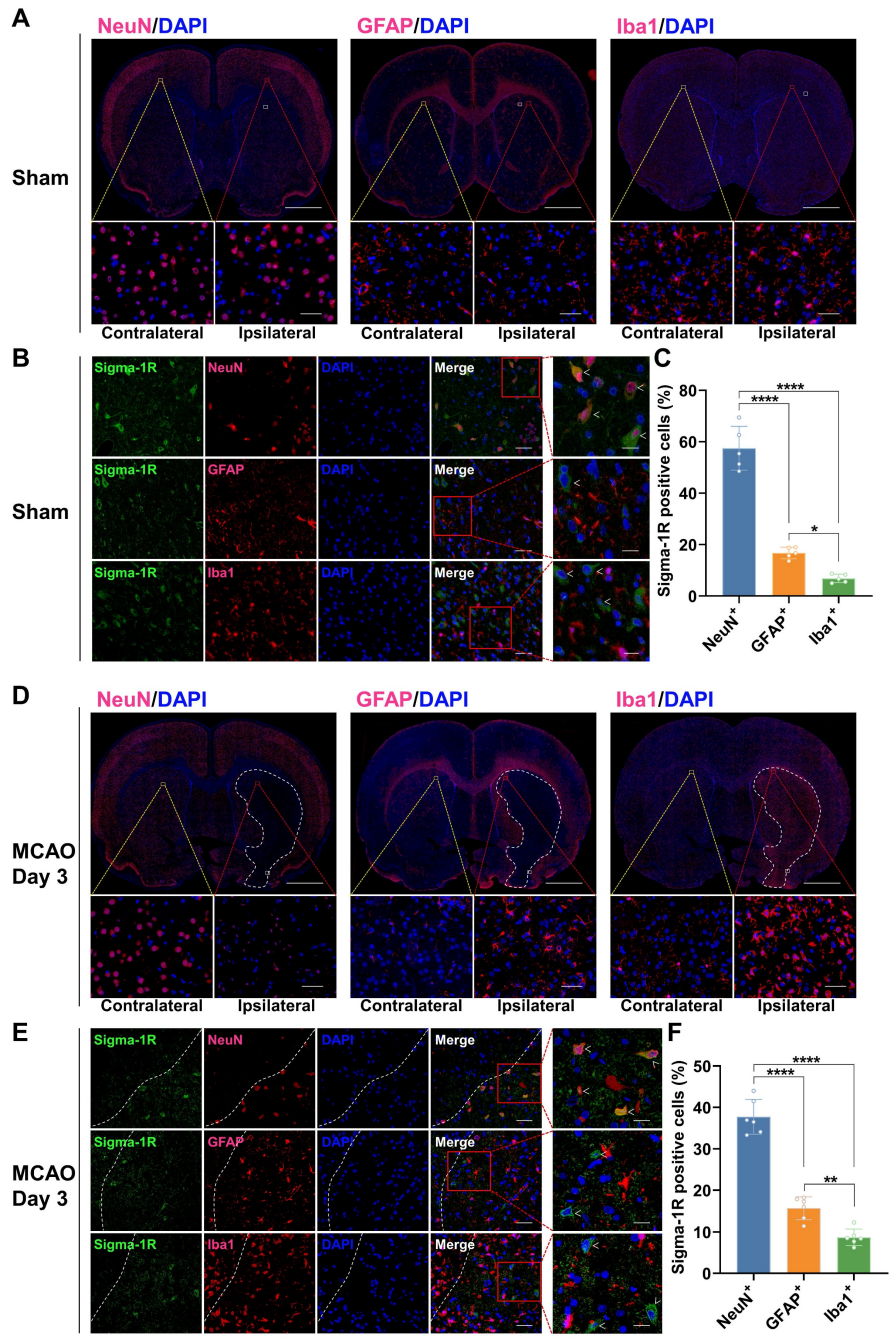
** Co-localization of sigma-1R with NeuN in rats of the sham group and MCAO group on day 3 post-stroke.** (A, D) Immunofluorescence images of NeuN, GFAP, and Iba1 in the ipsilateral versus contralateral hemispheres of rats from the sham group (A) and the MCAO group on day 3 post-stroke (D). The white dashed line outlines the approximate infarct area. The yellow box indicates the magnified region in the contralateral hemisphere, the red box indicates the magnified region in the ipsilateral hemisphere, and the white box corresponds to the enlarged views shown in panels B and E. Scale bars, 2 mm or 50 μm. (B, E) Immunofluorescence images illustrate the co-localization of sigma-1R (green) with NeuN-positive neurons (red) in the ipsilateral hemisphere of rats. White arrows indicate sigma-1R^+^ cells. Scale bars, 50 μm or 20 μm. (C, F) Quantification of sigma-1R^+^ neurons, astrocytes, and microglia. Values are mean ± SD (n = 5~6/group). *P < 0.05, **P < 0.01, and ****P < 0.0001.

**Figure 7:**
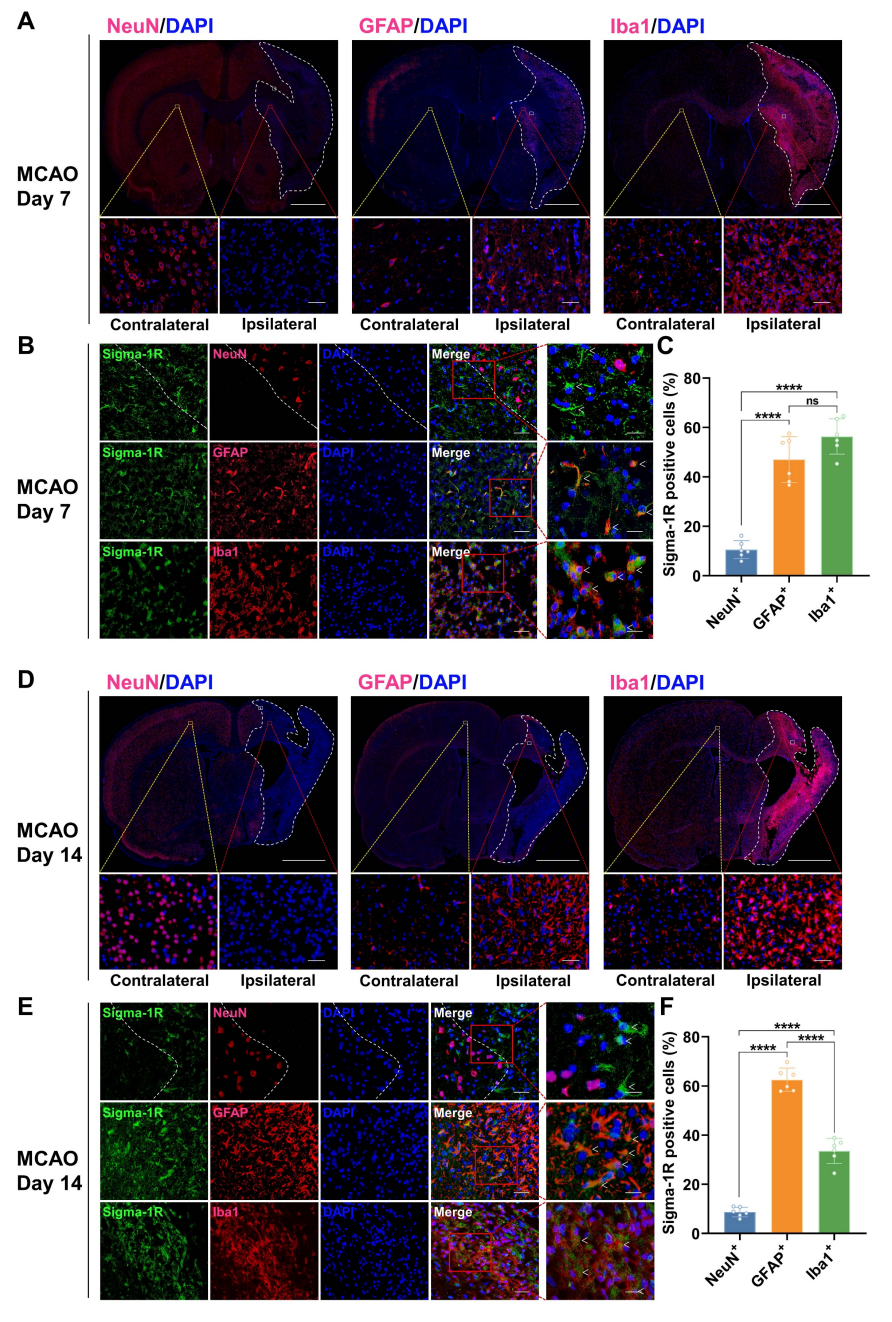
** Co-localization of sigma-1R with GFAP and Iba1 in MCAO rats on days 7 and 14 post-stroke.** (A, D) Immunofluorescence images of NeuN, GFAP, and Iba1 in the ipsilateral versus contralateral hemispheres of MCAO rats on days 7 (A) and 14 (D) post-stroke. The white dashed line outlines the approximate infarct area. The yellow box indicates the magnified region in the contralateral hemisphere, the red box indicates the magnified region in the ipsilateral hemisphere, and the white box corresponds to the enlarged views shown in panels B and E. Scale bars, 2 mm or 50 μm. (B, E) Immunofluorescence images illustrate the co-localization of sigma-1R (green) with GFAP-positive astrocytes (red) or Iba1-positive microglia (red) in the ipsilateral hemisphere of MCAO rats. White arrows indicate sigma-1R^+^ cells. Scale bars, 50 μm or 20 μm. (C, F) Quantification of sigma-1R^+^ neurons, astrocytes, and microglia. Values are mean ± SD (n = 6/group). ****P < 0.0001 and ns: no significance.

In the sham group, IF staining revealed that sigma-1R was primarily located in neurons (NeuN^+^), with less presence in astrocytes (GFAP^+^) and microglia (Iba1^+^) (Figure 6A-C). After stroke, the sigma-1R expression remained predominantly in neurons on day 3 (Figure 6D-F), and then shifted to microglia and astrocytes in the ischemic lesions on days 7 and 14 (Figure 7). Notably, the NeuN expression decreased significantly after stroke, while those of GFAP and Iba1 were elevated, indicating the death of neurons as well as the accumulation of astrocytes and microglia in the ischemic lesions (Figure 6D, Figure 7A, D, and Figure S3). These findings were corroborated by some datasets in the GEO database. GSE196737 dataset showed that microglia repopulation significantly increased the *Sigmar1* expression in microglia from both young and old mice (Figure S4B). Similarly, GSE35338 dataset revealed a significant reduction in *Sigmar1* expression in astrocytes in the MCAO group on day 3 post-stroke compared to that of the sham group (Figure S4C). Furthermore, IF staining showed that co-localization of sigma-1R with iNOS^+^ (pro-inflammatory/M1 microglia) or CD206^+^ (anti-inflammatory/M2 microglia) cells was relatively low on day 3. However, the number of Sigma-1R^+^CD206^+^ cells increased on day 14 (Figure S5). IF staining also revealed the co-localization of sigma-1R with 18 kDa Translocator Protein (TSPO) in glial cells on days 3, 7, 14, and 28 post-stroke, with the most prominent co-localization on days 7 and 14, and co-labeled cells still detectable on day 28 (Figure S6).

### Neurological outcomes, infarct area, [^18^F]FDG uptake in MCAO rats and their correlation with (*R*)-[^18^F]FBFP PET signals

Behavioral tests (Figure S7A-E) showed that MCAO rats had higher mNSS scores than the sham group immediately after stroke, and the mNSS scores gradually declined over time during recovery. The adhesive removal test revealed that MCAO rats took longer time to remove tape initially but were quicker on days 7, 14, 21, and 28. In the cylinder test, MCAO rats had significantly fewer contacts between their paretic forepaws and the cylinder wall than the sham group, especially on day 3, which improved during the later stages after stroke. Similarly, the corner test revealed increased right turns in MCAO rats, peaking on day 1 and declining over time. Overall, MCAO rats exhibited significant neurological deficits post-stroke, the impairments being most prominent in the acute phase and gradually alleviated thereafter. Further analysis revealed a significant correlation in MCAO rats on days 7, 14, and 21 post-stroke between (*R*)-[^18^F]FBFP PET signals and the behavioral results including the mNSS scores, the outcomes of adhesive removal test, cylinder test, and corner test (Figure S8). Figure S7F-H showed a correlation between body weight changes in MCAO rats and (*R*)-[^18^F]FBFP uptake on days 7, 14, and 21 post-stroke. In addition, [^18^F]FDG and (*R*)-[^18^F]FBFP PET images of MCAO rats were shown in Figure S9, and (*R*)-[^18^F]FBFP uptake correlated with [^18^F]FDG uptake on days 3 and 7 post-MCAO. (*R*)-[^18^F]FBFP uptake (the ipsilateral-to-contralateral SUV_mean_ ratio) on day 3 correlated with uptake on day 7 (Figure S9E, I), suggesting that early (*R*)-[^18^F]FBFP uptake may predict subsequent changes in sigma-1R expression. Nissl staining exhibited increased abnormal neurons and atrophied cell bodies in the MCAO group compared to the sham group. Notably, the ischemic lesion area did not change significantly over the studied duration, with no correlation between infarct size and (*R*)-[^18^F]FBFP uptake (Figure S10).

### Treatment with rtPA affected the uptake of (*R*)-[^18^F]FBFP in MCAO rats

**Figure 8:**
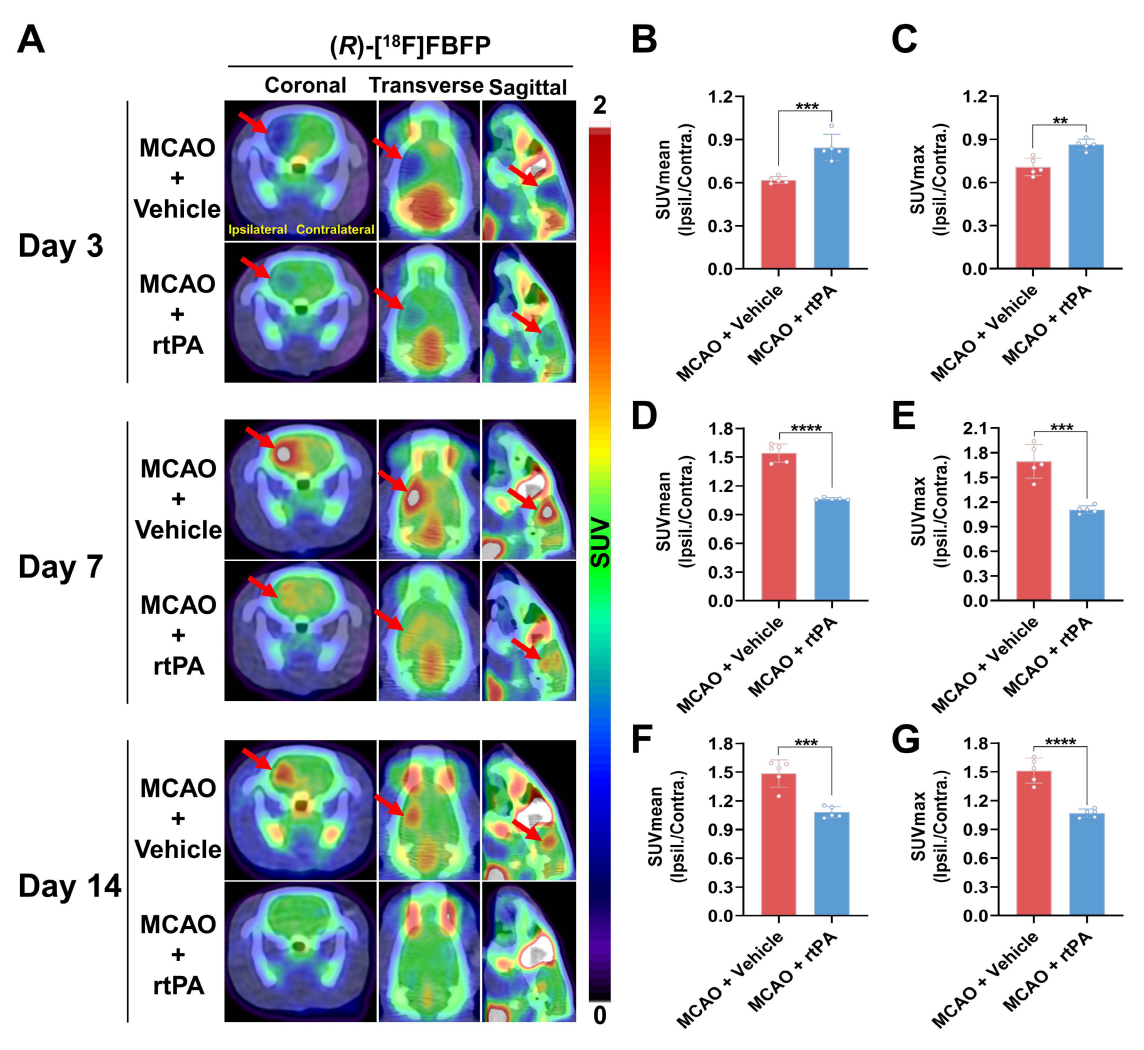
** Comparison of (*R*)-[^18^F]FBFP PET signals between MCAO rats with and without rtPA treatment.** (A) (*R*)-[^18^F]FBFP PET/CT images of the brains of MCAO rats with and without rtPA treatment on days 3, 7, and 14 post-stroke. Red arrows highlight the ischemic lesions in the ipsilateral hemisphere. (B-G) Quantification of the ratios of (*R*)-[^18^F]FBFP uptake (SUV_mean_ in B, D, F, and SUV_max_ in C, E, G) in the ipsilateral hemisphere relative to the contralateral hemisphere on days 3, 7, and 14 post-stroke. Values are mean ± SD (n = 5/group). **P < 0.01, ***P < 0.001, and ****P < 0.0001. MCAO, middle cerebral artery occlusion; rtPA, recombinant tissue plasminogen activator; SUV, standardized uptake value.

MCAO rats treated with 3 mg/kg rtPA or saline underwent longitudinal (*R*)-[^18^F]FBFP PET/CT imaging. In the MCAO + rtPA group, the ipsilateral-to-contralateral PET signal ratios were significantly higher on days 1 (Figure S11A-C) and 3 (Figure 8A-C) but lower on days 7 (Figure 8D-E), 14 (Figure 8F-G), and 21 (Figure S11D-E) post-stroke, compared to the MCAO + Vehicle group. On day 28, the PET signal ratios in the MCAO + rtPA group were significantly higher than in the MCAO + Vehicle group (Figure S11F-G). These results implied that rtPA treatment mitigated the difference in (*R*)-[^18^F]FBFP uptake between the affected and unaffected hemispheres of MCAO rats. Furthermore, in the MCAO + rtPA group, GFAP expression was reduced on day 28 post-stroke, indicating attenuated reactive astrogliosis. TGF-β in the lesion also decreased following rtPA treatment (Figure S12). These findings reflected the treatment-related modulation of the post-ischemic inflammatory/reparative response.

## Discussion

In this study, we used (*R*)-[^18^F]FBFP PET/CT imaging to investigate the spatiotemporal alterations in sigma-1R during experimental stroke progression. We further explored the correlation between (*R*)-[^18^F]FBFP uptake and neurological outcomes in ischemic stroke rats, as well as the impact of rtPA treatment on sigma-1R.

Ischemic stroke has high morbidity, disability, and mortality rates, with limited treatments, highlighting the urgent need for novel therapeutic targets 1-3. Notably, sigma-1R shows neuroprotective effects in ischemic stroke, protecting brain tissue from ischemia and aiding motor functional recovery 7, 9-13. Elucidating the time-dependent changes in sigma-1R after stroke is essential for gaining insights into this newly identified therapeutic target. An optimal radiotracer allows real-time visualization of cerebral sigma-1R, aiding in unraveling its mystery in ischemic stroke and supporting related drug development. Consequently, we innovatively selected the novel radiotracer (*R*)-[^18^F]FBFP to evaluate sigma-1R in animal stroke models.

Herein, we demonstrated that (*R*)-[^18^F]FBFP effectively detected sigma-1R changes in the infarct regions of MCAO rats, with excellent stability and binding specificity. HPLC analysis confirmed its RCP and stability (Figure S1). The 90-minute PET scan exhibited distinct (*R*)-[^18^F]FBFP uptake in the rat brain, with varying signals in the ischemic and unaffected hemispheres (Figure 2). TACs matched previous findings 19. Additionally, SA4503 blocking significantly reduced (R)-[^18^F]FBFP accumulation in MCAO rats, confirming its specific binding to sigma-1R, in line with prior research (Figure 4) 19. PET imaging revealed that (*R*)-[^18^F]FBFP uptake in the ischemic lesions decreased considerably in the acute phase (days 1 and 3), increased in the subacute phase (days 7 and 14), and then gradually declined in the chronic phase (days 21 and 28) (Figure 3). These *in vivo* findings were supported by *ex vivo* autoradiography (Figure S2) and IF staining of sigma-1R (Figure 5). It should be noted that samples for autoradiography and IF staining were not obtained from rats undergoing PET imaging, since PET imaging required longitudinal monitoring, whereas autoradiography and IF relied on frozen or paraffin-embedded sections. Nevertheless, results derived from the same experimental groups and time points exhibited consistent trends, which supported the reliability of our findings. Moreover, our analysis of the GSE36010 dataset 26 and previous studies 9, 29 using western blot and IF staining indicated reduced sigma-1R in the ischemic core on days 1 and 3 post-stroke, with increased expression on day 14, aligning with our PET imaging results.

IF staining revealed that the main expression of sigma-1R shifted from neurons to microglia and astrocytes after stroke, accompanied by numerous necrotic neurons, microglia infiltration, and astrocyte aggregation in ischemic lesions (Figure 6,7, Figure S3). This pattern of shifting was captured by PET imaging and corroborated well with previous studies using IF staining that sigma-1R was expressed in neurons during the acute phase and in reactive astrocytes during stroke recovery 9, 29. Studies have suggested that sigma-1R can reduce apoptosis and promote astrocyte survival, restraining neurodegeneration post-ischemia 12. Astrocytic sigma-1R deficiency worsens neuroinflammation and neuronal apoptosis by activating the NF-κB pathway 30. Thus, we speculate that the aggregation of sigma-1R^high^ astrocytes in ischemic lesions may alleviate neuroinflammation and neuronal damage, aiding stroke recovery. Although the GSE35338 dataset showed reduced astrocytic *Sigmar1* expression on day 3 but no change between days 3 and 7 28, its findings were based on mRNA transcription and therefore may differ from the protein expression revealed by PET imaging. Additionally, a subsequent study confirmed the localization of sigma-1R in microglia 8. In mice with cortical stab injuries, activated microglia and astrocytes exhibited higher sigma-1R levels than those in healthy tissues 8. Our analysis of the GSE196737 dataset revealed that microglia repopulation could increase *Sigmar1* levels in microglia 27. These results matched our findings of elevated sigma-1R expression in microglia in the ischemic lesions.

In addition, IF staining revealed that sigma-1R expression may associate with distinct inflammatory phenotypes at different stages after stroke. Specifically, on day 3 post-stroke, sigma-1R showed limited co-localization with either iNOS^+^ or CD206^+^ microglia/macrophages. On day 14, instead, its co-localization with CD206^+^ cells was markedly increased, indicating a preferential association with anti-inflammatory/reparative phenotypes during the recovery phase (Figure S5). This was relevant to the interpretation of our PET findings. The prolonged presence of TSPO^+^ and sigma-1R^+^ double-positive cells, particularly during the subacute phase of stroke (days 7 and 14), implied that sigma-1R may modulate not only early neuroinflammation but also later reparative processes (Figure S6). Together with the observed increase in GFAP^+^ astrocytes, these findings suggested that the elevated PET signal may reflect the combined influence of anti-inflammatory and reparative microglia/astrocytes, rather than a unidirectional pro-inflammatory process. Due to experimental constraints, TSPO PET imaging was not performed; however, future studies incorporating this *in vivo* approach will be valuable for elucidating the temporal interplay between sigma-1R expression and neuroinflammation.

Further behavioral tests revealed a significant correlation between neurological outcomes during stroke recovery and (*R*)-[^18^F]FBFP uptake (Figure S7, 8). This discovery aligned with prior findings that sigma-1R activation ameliorated stroke-induced neurological dysfunction 6, 9. Sigma-1R activation is vital for Ca^2+^ regulation and stress response, inhibiting ER stress-induced neuronal apoptosis after cerebral ischemia/reperfusion injury 6, 11. It promotes brain-derived neurotrophic factor (BDNF) secretion and neurite outgrowth, thereby enhancing neural plasticity and improving stroke outcomes 9, 10, 31. Additionally, sigma-1R plays a neuroprotective role in ischemic stroke by regulating microglial activation, reducing neuroinflammation, and aiding functional recovery through efferocytosis of infiltrating macrophages/microglia 13, 32. Studies in other CNS diseases have found that sigma-1R modulation enhances autophagy and M1/M2 microglia polarization, and ameliorates NF-κB-induced neuroinflammation 30, 33, 34. Sigma-1R activation also enhances pericyte survival, alleviates blood-brain barrier disruption, and promotes oligodendrogenesis and white-matter functional recovery post-stroke 35-37. These mechanisms elucidate the impact of sigma-1R on stroke recovery and offer potential explanations for the association between (*R*)-[^18^F]FBFP uptake and neurological outcomes.

In addition, (*R*)-[^18^F]FBFP uptake correlated with [^18^F]FDG uptake on days 3 and 7 post-stroke (Figure S9), suggesting that regional sigma-1R expression was linked to metabolic activity. This was consistent with previous findings that sigma-1R modulated cellular stress responses, mitochondrial function, and metabolism 5. (*R*)-[^18^F]FBFP uptake on day 3 correlated with uptake on day 7, providing preliminary evidence that early (*R*)-[^18^F]FBFP uptake may influence or reflect subsequent changes in sigma-1R in recovery processes. The complementary use of (*R*)-[^18^F]FBFP and [^18^F]FDG PET may provide a more comprehensive understanding of the complex pathophysiological changes after stroke. However, the relatively low correlation coefficients and marginal significance indicated that these associations were weak and should be interpreted with caution. Further studies with larger cohorts are warranted to establish the reliability and mechanistic implications of these correlations. Notably, the infarct area shown by Nissl staining did not correlate with (*R*)-[^18^F]FBFP uptake, probably because the infiltration of glial cells with high expression of sigma-1R compensated for that lost due to neuronal death (Figure S10). While (*R*)-[^18^F]FBFP is not an ideal marker for neuronal loss, it is valuable for predicting post-stroke neurological recovery and offering a promising therapeutic target.

Intravenous rtPA is commonly recommended for acute ischemic stroke management. Our study found that rtPA modulated sigma-1R expression in ischemic lesions, shown by reduced differences in (*R*)-[^18^F]FBFP uptake between the diseased and unaffected hemispheres over time (Figure 8 and Figure S11). This indicated that (*R*)-[^18^F]FBFP PET imaging effectively evaluated the thrombolytic effects post-stroke. rtPA can improve stroke outcomes by facilitating early vessel recanalization and reducing ischemic damage and necrosis 38, which may alter sigma-1R expression. Moreover, reduced GFAP indicated attenuated astrogliosis after rtPA, whereas decreased TGF-β may reflect complex temporal dynamics, reduced injury burden, or altered remodeling rather than a simple loss of reparative signaling (Figure S12). However, owing to the limited availability of well-matched tissue, this finding should be taken with caution. Quantitative analyses, including expanded phenotypic markers and TSPO PET, would be warranted to clarify how rtPA modulates inflammation and to link these changes more directly to (*R*)-[^18^F]FBFP uptake, and the intricate mechanisms by which rtPA affects sigma-1R remain to be elucidated.

This study has several limitations. First, we did not pursue the molecular mechanism by which sigma-1R was modulated post-stroke, since it would be out-of-scope for the development of sigma-1R-targeting PET tracer. Second, the dynamic changes of sigma-1R in MCAO rats would need further validation in patients with stroke. Nevertheless, our study establishes the feasibility of using (*R*)-[^18^F]FBFP PET imaging for non-invasive visualization of sigma-1R alterations after stroke and provides valuable insight into the response to rtPA treatment. Our study highlights the therapeutic potential of sigma-1R as a neuroprotective target for mitigating stroke-induced cellular damage.

## Conclusion

(*R*)-[^18^F]FBFP enabled a non-invasive real-time tracking of sigma-1R alterations in the rat brain after stroke, demonstrating excellent specificity and stability. (*R*)-[^18^F]FBFP uptake strongly correlated with neurological outcomes during stroke recovery. Post-stroke changes in sigma-1R were impacted by neuronal loss and glial cell accumulation. The therapeutic efficacy of rtPA could be monitored with (*R*)-[^18^F]FBFP PET imaging. Collectively, this investigation employs (*R*)-[^18^F]FBFP to deepen understanding of the role of sigma-1R, an attractive therapeutic target, in stroke. This will lay a foundation for further elucidating the underlying therapeutic mechanisms of sigma-1R, advancing related drug development and clinical application of this radiotracer.

## Supplementary Material

Supplementary figures and tables.

## Abbreviations

BDNF: brain-derived neurotrophic factor
CNS: central nervous system
CT: computed tomography
ER: endoplasmic reticulum
GEO: Gene Expression Omnibus
HPLC: high-performance liquid chromatography
IF: immunofluorescence
MAM: mitochondria-associated endoplasmic reticulum membrane
MCA: middle cerebral artery
MCAO: middle cerebral artery occlusion
mNSS: modified neurological severity score
PBS: phosphate-buffered saline
PET: positron emission tomography
RCP: radiochemical purity
RCY: radiochemical yield
rtPA: recombinant tissue plasminogen activator
Sigma-1R: sigma-1 receptor
SUV: standardized uptake value
TAC: time-activity curve
TSPO: 18 kDa Translocator Protein
[^18^F]FBFP: 1-(4-[^18^F]Fluorobenzyl)-4-[(tetrahydrofuran-2-yl)methyl]piperazine
