# Supplementary material for: Longitudinal in vivo (R)-[18F]FBFP PET imaging for preclinical evaluation of cerebral sigma-1 receptor after ischemic stroke
Source: Theranostics. 2026 Jan 1;16(2):970–85. doi: 10.7150/thno.117418 (PMC12675007; doi:10.7150/thno.117418)
Supplement: Supplementary file 1 — Supplementary figures and tables. [file thnov16p0970s1.pdf]

Supplemental Data

Original article

Longitudinal *in vivo* (*R*)-[^{18}F]FBFP PET imaging for preclinical evaluation of cerebral sigma-1 receptor after ischemic stroke

Jingfei Yang[1, 2#], Luoxia Liu[1, 2#], Huimin Zhou[1, 2], Chuanzhi Huang[1, 2], Dongdong Wang[1, 2], Ziqiang Wang[1, 2], Yifan Shi[1, 2], Shuang Song[1, 2], Xiaoyun Deng[1, 2], Yuankai Zhu[1, 2], Jun Zhao[1, 2], Zhouping Tang[2, 3]*, Hongmei Jia[4]*, Xiaohua Zhu[1, 2, 5]*

[#] Jingfei Yang and Luoxia Liu contributed equally to this work.

[1] Department of Nuclear Medicine, Tongji Hospital, Tongji Medical College, Huazhong University of Science and Technology. Wuhan 430030, China.

[2] National Medical Center for Major Public Health Events. Wuhan 430030, China.

[3] Department of Neurology, Tongji Hospital, Tongji Medical College, Huazhong University of Science and Technology. Wuhan 430030, China.

[4] Key Laboratory of Radiopharmaceuticals (Beijing Normal University), Ministry of Education, College of Chemistry, Beijing Normal University. Beijing 100875, China.

[5] State Key Laboratory for Diagnosis and Treatment of Severe Zoonotic Infectious Disease. Wuhan 430030, China.

***Corresponding Author**

Xiaohua Zhu, MD, PhD. (Email: evazhu@tjh.tjmu.edu.cn; Tel: +86-27-83665157; Fax: +86-27-83665157.)

Hongmei Jia, PhD. (Email: hmjia@bnu.edu.cn)

Zhouping Tang, MD, PhD. (Email: ddjtzp@163.com)

1. Material and Methods

1.1 Quality control

Quality control was executed on an analytical HPLC column (Symmetry C18 Column, 5 μm, 4.6 mm × 250 mm; Waters Corporation, USA) at a flow rate of 1 mL/min, using 50% water and 50% acetonitrile as the mobile phase, each with 0.1% triethylamine. The radiochemical purity (RCP) and molar activity of (*R*)-[^{18}F]FBFP were analyzed by HPLC.

1.2 *In vitro* stability studies

The *in vitro* stability of (*R*)-[^{18}F]FBFP in serum and phosphate-buffered saline (PBS, Servicebio, China) was determined by measuring the RCP at various time points at 37 °C. The (*R*)-[^{18}F]FBFP solution was incubated in either serum or PBS at 37 °C for 1, 2, and 4 h, then 50 μL of the sample was mixed with an equal volume of acetonitrile and centrifuged at 5000 rpm for 10 min. The supernatant was measured by the analytical radio-HPLC (Symmetry C18 Column, 5 μm, 4.6 mm × 250 mm; Waters Corporation, USA) at a flow rate of 1 mL/min. The mobile phase consisted of a 50:50 mixture of water and acetonitrile, each containing 0.1% triethylamine.

1.3 TTC staining

On day 3 post-stroke, the rats (n: 4 × 2 = 8) were euthanized, and their brains were swiftly excised and stored at -20 °C for 20 min. Coronal sections were then prepared at 2-mm intervals and incubated

for 15 min at 37 °C in a 2% solution of 2,3,5-triphenyl tetrazolium chloride (TTC, Servicebio, China). Image analysis was performed by ImageJ software (National Institutes of Health, Bethesda, Maryland). The infarct volume was calculated by summing the infarcted areas from all sections and multiplying by the thickness of each slice. The results were quantified as the percentage of infarct volume relative to the total volume of the ipsilateral hemisphere.

1.4 Behavioral tests

Modified neurological severity score (mNSS)

Eight rats per group were subjected to behavioral tests on days 1, 3, 7, 14, 21, and 28 after stroke. The mNSS encompassed a composite assessment of motor abilities, sensory disturbances, reflexes, and balance tests. Neurological deficits were graded on a scale from 0 to 18, where 0 represented normal function and 18 indicated the most severe deficit. Higher scores reflected more significant neurological impairment. The severity of the injury was categorized into three levels: mild (scores 1-6), moderate (scores 7-12), and severe (scores 13-18).

Adhesive removal test

Before beginning the experiment, animal cages were positioned in the test chamber for at least 30 min to allow the rats to acclimate to the new environment. Following this acclimation period, each rat was gently placed into a new cage for an additional 60 s to further enhance their comfort and reduce stress. Adhesive tapes were applied to the hairless areas of the forepaws using forceps and the rats were returned to the home cage. The time taken for the animal to contact and remove each adhesive tape was recorded, with a maximum allowable time of 120 s. If the adhesive tape was not removed by the rat within this timeframe, the experimenter intervened to remove the tape, documenting 120 s as the recorded

time.

Cylinder test

Each rat was placed individually into a transparent cylinder (diameter: 20 cm, height: 30 cm) for 3 min. During this time, the number of times each forepaw contacted the cylinder wall was counted. The score was defined using the following formula: [(the number of contacts with the non-paretic limb) - (the number of contacts with the paretic limb)] / (total number of contacts).

Corner test

Each rat was positioned between two boards set at a 30° angle with a small opening at the end. As the rat entered deeply into the corner, the two boards stimulated both sides of its vibrissae. Subsequently, the rat reared forward and upward before turning back to face the open end. MCAO rats demonstrated a significant preference for turning to the right because of a loss of vibrissae sensation and an accompanying disability in rearing to the left. Each rat underwent ten trials, and the direction of turning (left or right) was recorded.

1.5 Nissl staining

Using Nissl staining, we assessed neuronal integrity by measuring Nissl bodies in the neuron cytoplasm. For Nissl staining, after the brain sections were dewaxed and rehydrated, they were placed in a 1% toluidine blue solution at 56 °C for 20 min, and washed with distilled water to remove excess stain. Subsequently, the sections were gradually dehydrated using a series of ethanol solutions in increasing concentrations (70%, 80%, 95%, and finally 100%). After dehydration, the sections were immersed in 100% xylene to facilitate the clearing process. To preserve the sections, the cover glass was securely sealed using neutral balsam. Finally, the sections were imaged under a microscope (Olympus CX-31,

Tokyo, Japan), and images were analyzed using ImageJ software (National Institutes of Health, Bethesda, Maryland) to assess the extent of Nissl body damage.

1.6 Immunohistochemistry

Brain sections from MCAO + Vehicle and MCAO + rtPA groups were subjected to immunohistochemical staining for GFAP and TGF-β. Briefly, paraffin-embedded coronal sections were deparaffinized, rehydrated, and subjected to antigen retrieval. Endogenous peroxidase activity was quenched with 3% H_2O_2, and non-specific binding was blocked with 5% normal goat serum. Sections were incubated overnight at 4 °C with primary antibodies anti-GFAP (1:500, Cat#ab68428, Abcam, UK) and anti-TGF-β (1:100, Cat#bs-0086R, Bioss, China), followed by incubation with HRP-conjugated secondary antibodies. Immunoreactivity was visualized using DAB substrate and counterstained with hematoxylin. Images were acquired under a light microscope.

2. Supplemental tables

Table S1. The brain uptake of (*R*)-[^{18}F]FBFP in MCAO rats.

	Group	SUV$_{mean}$	SUV$_{mean}$ (Ipsilateral/ Contralateral)	SUV$_{max}$	SUV$_{max}$ (Ipsilateral/ Contralateral)
**(*R*)-[^{18}F]FBFP PET/CT**	Sham	Ipsilateral: 1.08 ± 0.17; Contralateral: 1.08 ± 0.17 (*P* = 0.9851[1])	1.00 ± 0.03	Ipsilateral: 1.52 ± 0.19; Contralateral: 1.49 ± 0.19 (*P* = 0.7405[1])	1.03 ± 0.10
	MCAO on Day 1	Ipsilateral: 0.81 ± 0.15; Contralateral: 1.15 ± 0.23 (*P* = 0.0027[1]*)	0.74 ± 0.13 (*P* < 0.0001[2]*)	Ipsilateral: 1.10 ± 0.23; Contralateral: 1.49 ± 0.28 (*P* = 0.0048[1]*)	0.76 ± 0.07 (*P* < 0.0001[2]*)
	MCAO on Day 3	Ipsilateral: 0.77 ± 0.15; Contralateral: 1.14 ± 0.36 (*P* = 0.0172[1]*)	0.63 ± 0.09 (*P* < 0.0001[2]*)	Ipsilateral: 1.06 ± 0.29; Contralateral: 1.55 ± 0.27 (*P* = 0.0190[1]*)	0.76 ± 0.05 (*P* < 0.0001[2]*)
	MCAO on Day 7	Ipsilateral: 1.68 ± 0.26; Contralateral: 1.12 ± 0.26 (*P* = 0.0002[1]*)	1.74 ± 0.23 (*P* < 0.0001[2]*)	Ipsilateral: 2.17 ± 0.15; Contralateral: 1.47 ± 0.19 (*P* < 0.0001[1]*)	1.89 ± 0.35 (*P* < 0.0001[2]*)
	MCAO on Day 14	Ipsilateral: 1.50 ± 0.32; Contralateral: 1.09 ± 0.24 (*P* = 0.0115[1]*)	1.42 ± 0.23 (*P* < 0.0001[2]*)	Ipsilateral: 1.97 ± 0.27; Contralateral: 1.52 ± 0.18 (*P* = 0.0028[1]*)	1.54 ± 0.31 (*P* = 0.0003[2]*)

	MCAO on Day 21	Ipsilateral: 1.22 ± 0.33; Contralateral: 1.16 ± 0.37 (*P* = 0.7620[1])	1.01 ± 0.21 (*P* = 0.2224[2])	Ipsilateral: 1.66 ± 0.25; Contralateral: 1.52 ± 0.32 (*P* = 0.3701[1])	1.09 ± 0.17 (*P* = 0.2973[2])
	MCAO on Day 28	Ipsilateral: 0.83 ± 0.17; Contralateral: 1.11 ± 0.25 (*P* = 0.0306[1]*)	0.74 ± 0.10 (*P* < 0.0001[2]*)	Ipsilateral: 1.09 ± 0.25; Contralateral: 1.47 ± 0.19 (*P* = 0.0239[1]*)	0.79 ± 0.03 (*P* < 0.0001[2]*)
**Blocking study**	Sham (Baseline)	Ipsilateral: 1.05 ± 0.22; Contralateral: 1.06 ± 0.21		Ipsilateral: 1.49 ± 0.18; Contralateral: 1.51 ± 0.14	
	Sham (Blocking)	Ipsilateral: 0.30 ± 0.02 (*P* = 0.0005[3]*); Contralateral: 0.28 ± 0.04 (*P* = 0.0003[3]*)		Ipsilateral: 0.51 ± 0.03 (*P* < 0.0001[3]*); Contralateral: 0.50 ± 0.04 (*P* < 0.0001[3]*)	
	MCAO on Day 7 (Baseline)	Ipsilateral: 1.61 ± 0.33; Contralateral: 1.01 ± 0.15		Ipsilateral: 2.07 ± 0.23; Contralateral: 1.48 ± 0.14	
	MCAO on Day 7 (Blocking)	Ipsilateral: 0.42 ± 0.08 (*P* < 0.0001[3]*); Contralateral: 0.31 ± 0.06 (*P* < 0.0001[3]*)		Ipsilateral: 0.65 ± 0.04 (*P* < 0.0001[3]*); Contralateral: 0.56 ± 0.07 (*P* < 0.0001[3]*)	
	MCAO on Day 14 (Baseline)	Ipsilateral: 1.44 ± 0.23; Contralateral: 1.04 ± 0.28		Ipsilateral: 1.97 ± 0.12; Contralateral: 1.44 ± 0.20	
	MCAO on Day 14 (Blocking)	Ipsilateral: 0.49 ± 0.06 (*P* < 0.0001[3]*); Contralateral: 0.39 ± 0.06 (*P* = 0.0009[3]*)		Ipsilateral: 0.70 ± 0.08 (*P* < 0.0001[3]*); Contralateral: 0.54 ± 0.04 (*P* = 0.0079[3]*)	
**(*R*)-**	MCAO + Vehicle on Day 1		0.81 ± 0.02		0.84 ± 0.06

**[^{18}F]FBFP PET/CT**	MCAO + rtPA on Day 1	0.87 ± 0.05 (*P* = 0.0323[4]*)	0.91 ± 0.03 (*P* = 0.0375[4]*)
**(*R*)-[^{18}F]FBFP PET/CT**	MCAO + Vehicle on Day 3	0.62 ± 0.02	0.71 ± 0.06
	MCAO + rtPA on Day 3	0.85 ± 0.09 (*P* = 0.0007[4]*)	0.86 ± 0.04 (*P* = 0.0013[4]*)
**(*R*)-[^{18}F]FBFP PET/CT**	MCAO + Vehicle on Day 7	1.54 ± 0.09	1.70 ± 0.21
	MCAO + rtPA on Day 7	1.06 ± 0.01 (*P* < 0.0001[4]*)	1.11 ± 0.05 (*P* = 0.0002[4]*)
**(*R*)-[^{18}F]FBFP PET/CT**	MCAO + Vehicle on Day 14	1.49 ± 0.14	1.51 ± 0.13
	MCAO + rtPA on Day 14	1.08 ± 0.06 (*P* = 0.0004[4]*)	1.07 ± 0.04 (*P* < 0.0001[4]*)
**(*R*)-[^{18}F]FBFP PET/CT**	MCAO + Vehicle on Day 21	1.14 ± 0.05	1.14 ± 0.06
	MCAO + rtPA on Day 21	1.04 ± 0.05 (*P* = 0.0101[4]*)	1.00 ± 0.04 (*P* = 0.0025[4]*)
**(*R*)-[^{18}F]FBFP PET/CT**	MCAO + Vehicle on Day 28	0.93 ± 0.06	0.93 ± 0.08
	MCAO + rtPA on Day 28	1.05 ± 0.02 (*P* = 0.0052[4]*)	1.04 ± 0.04 (*P* = 0.0187[4]*)

[1]Ipsilateral hemisphere vs. Contralateral hemisphere; [2]Compare to the sham group; [3]Baseline condition vs. Blocking condition; [4] MCAO + Vehicle group vs. MCAO + rtPA group. Values are mean ± SD. *$P < 0.05$. MCAO, middle cerebral artery occlusion; rtPA, recombinant tissue plasminogen activator; SUV, standardized uptake value.

Table S2. Changes in sigma-1R expression after ischemic stroke assessed by immunofluorescence staining.

	Group	Sigma-1R$^+$ cells per field (%, Ipsilateral)	Sigma-1R$^+$ cells per field (%, Contralateral)	*P* value (Ipsilateral vs. Contralateral)
Immunofluorescence staining	Sham group	49.23 ± 6.47	48.71 ± 6.82	0.9044
	MCAO group on Day 1	34.10 ± 4.83	49.41 ± 3.97	0.0006*
	MCAO group on Day 3	32.04 ± 5.00	49.82 ± 3.38	< 0.0001*
	MCAO group on Day 7	63.42 ± 8.16	49.76 ± 4.85	0.0055*
	MCAO group on Day 14	63.44 ± 3.06	51.04 ± 3.08	< 0.0001*
	MCAO group on Day 21	49.12 ± 7.31	48.05 ± 5.06	0.7943
	MCAO group on Day 28	36.51 ± 4.24	50.70 ± 2.32	0.0002*

Values are mean ± SD. *$P < 0.05$.

3. Supplemental figures

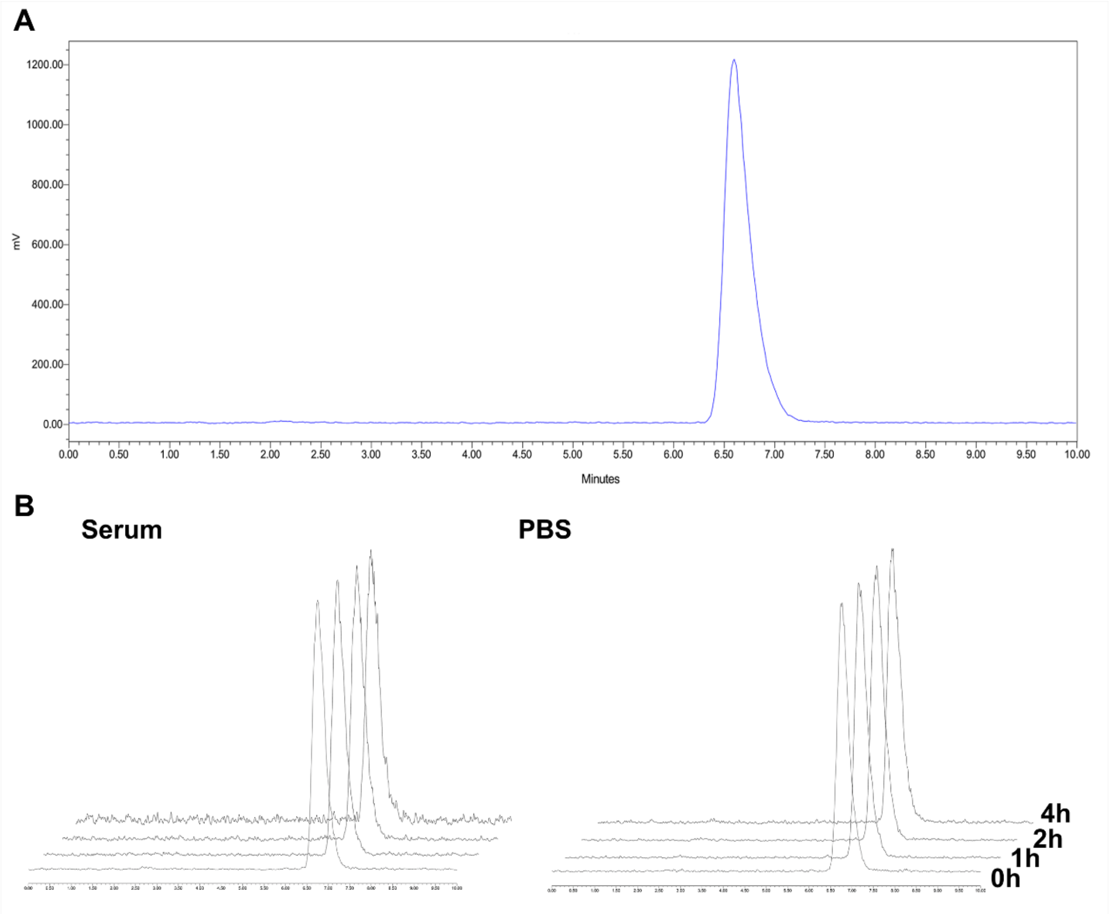

Figure S1. HPLC chromatograms of (*R*)-[^{18}F]FBFP.

(A) The radiochemical purity of (*R*)-[^{18}F]FBFP via radio-HPLC. (B) Analytical radio-HPLC chromatograms of (*R*)-[^{18}F]FBFP in serum or PBS incubated at 37 °C for 0, 1, 2, or 4h after synthesis. Conditions: CH_3CN/H_2O (containing 0.1% triethylamine) = 50/50, v/v, flow rate = 1 mL/min. PBS, phosphate-buffered saline.

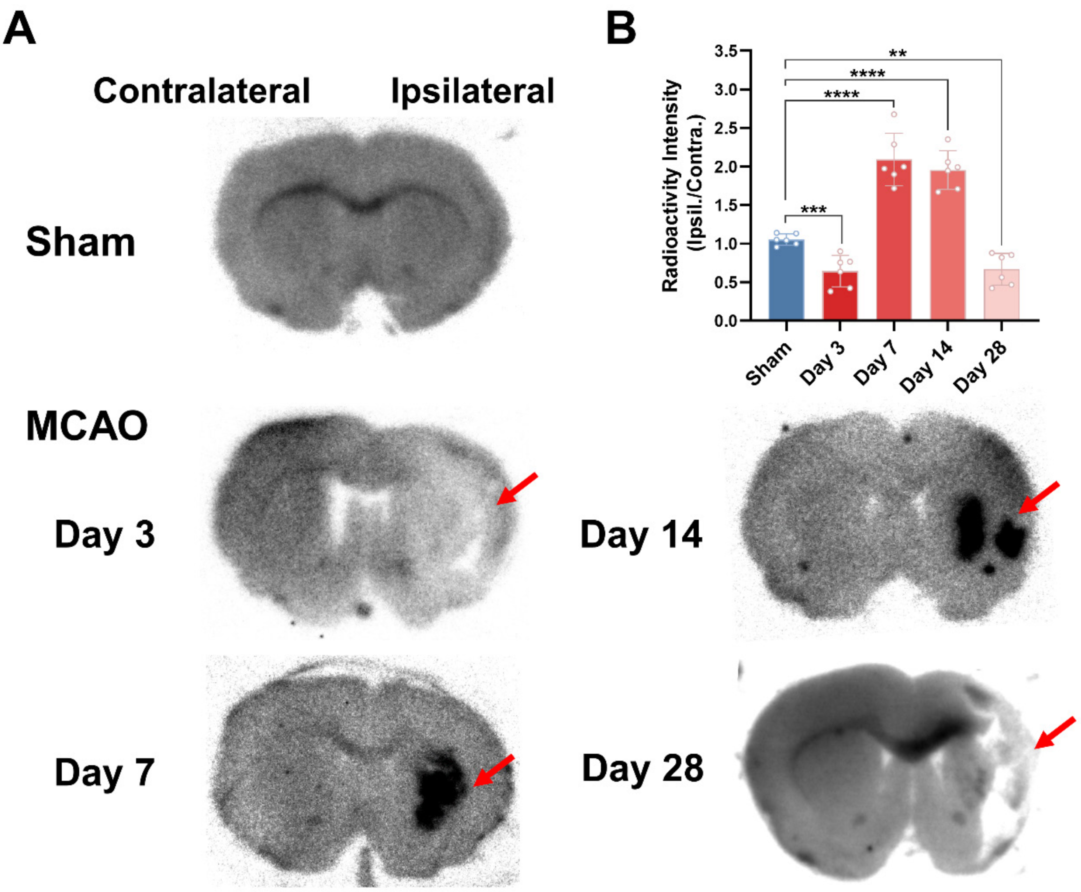

Figure S2. ***Ex vivo*** **autoradiography of (*R*)-[^{18}F]FBFP in rat brains after ischemic stroke.**

(A) Representative autoradiography images of the sham group and the MCAO group on days 3, 7, 14, and 28 after stroke. (B) Quantification of autoradiography studies with (*R*)-[^{18}F]FBFP. Values are mean ± SD (n = 6/group). Statistical significance was calculated with the one-way ANOVA test. $^{**}P < 0.01$, $^{***}P < 0.001$, and $^{****}P < 0.0001$. MCAO, middle cerebral artery occlusion.

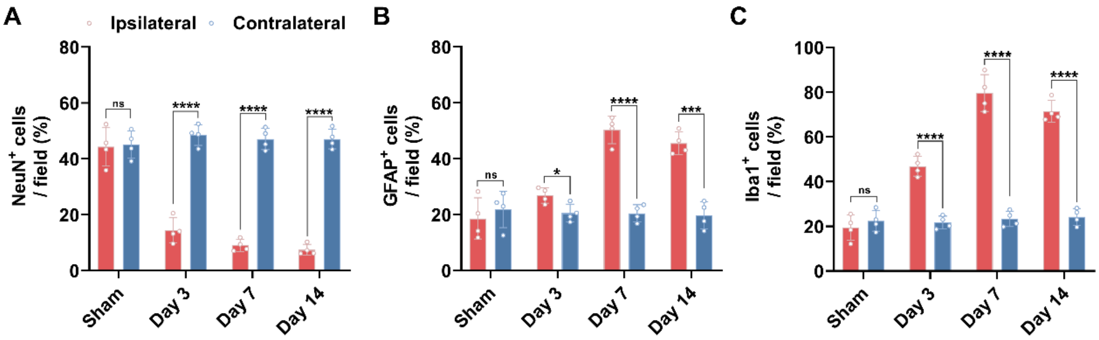

Figure S3. Changes in NeuN, GFAP, and Iba1 levels in rat brains after ischemic stroke.

Quantification of NeuN^{+} cells, GFAP^{+} cells, and Iba1^{+} cells per field. Values are mean ± SD (n = 4/group). *$P < 0.05$, ***$P < 0.001$, ****$P < 0.0001$, and ns: no significance.

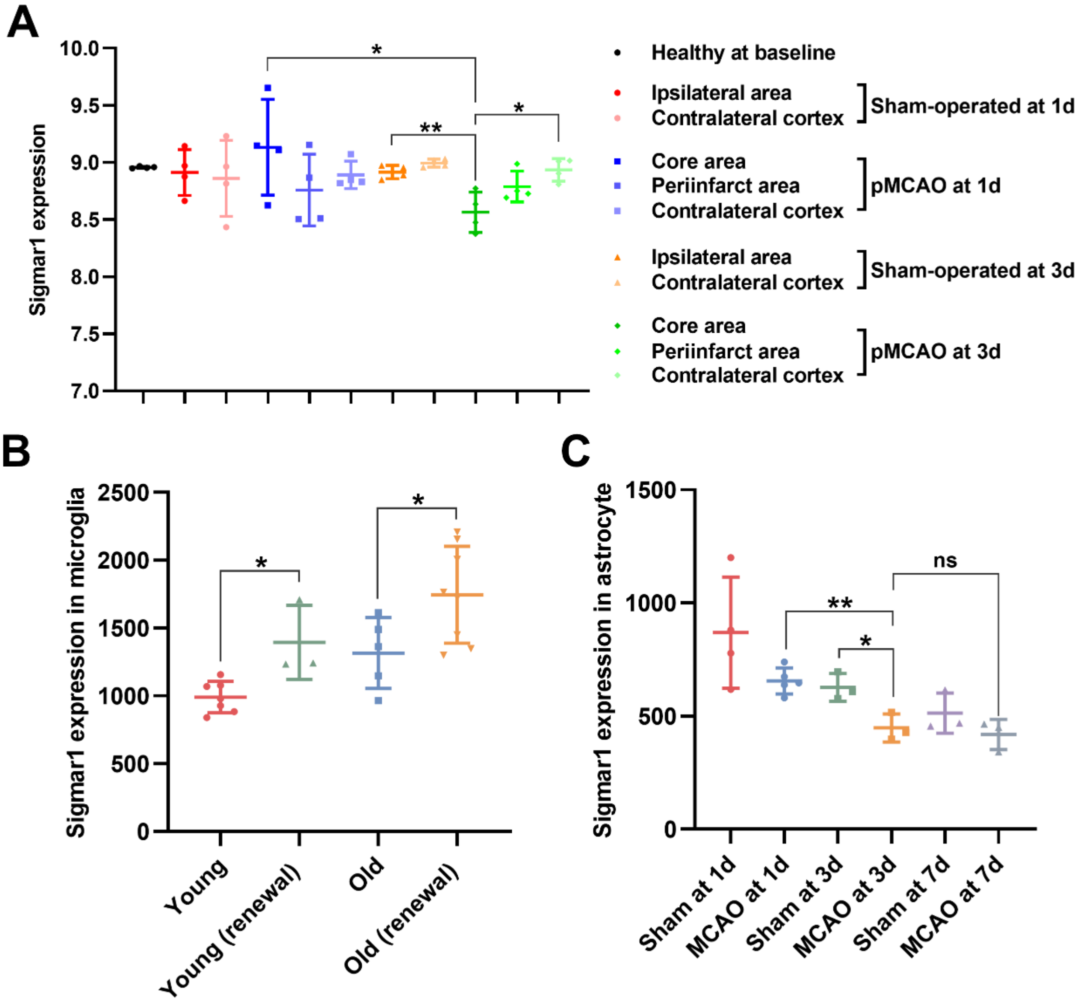

Figure S4. Differential expression analysis of *Sigmar1* in rats and mice with stroke from GEO datasets.

(A) mRNA expression of *Sigmar1* in brain tissues of rats based on data from the GSE36010 dataset (sham vs. permanent MCAO on days 1 and 3 post-stroke). (B) mRNA expression of *Sigmar1* in microglia from young and old mice, analyzed from the GSE196737 dataset (Microglia depletion vs. Microglia renewal). (C) mRNA expression of *Sigmar1* in astrocytes of mice, analyzed from the GSE35338 dataset (sham vs.

MCAO on days 1, 3, and 7 post-stroke). Values are mean ± SD. Statistical significance was calculated with the two-tailed unpaired Student's t-test and one-way ANOVA. *P < 0.05, **P < 0.01, and ns: no significance. MCAO, middle cerebral artery occlusion.

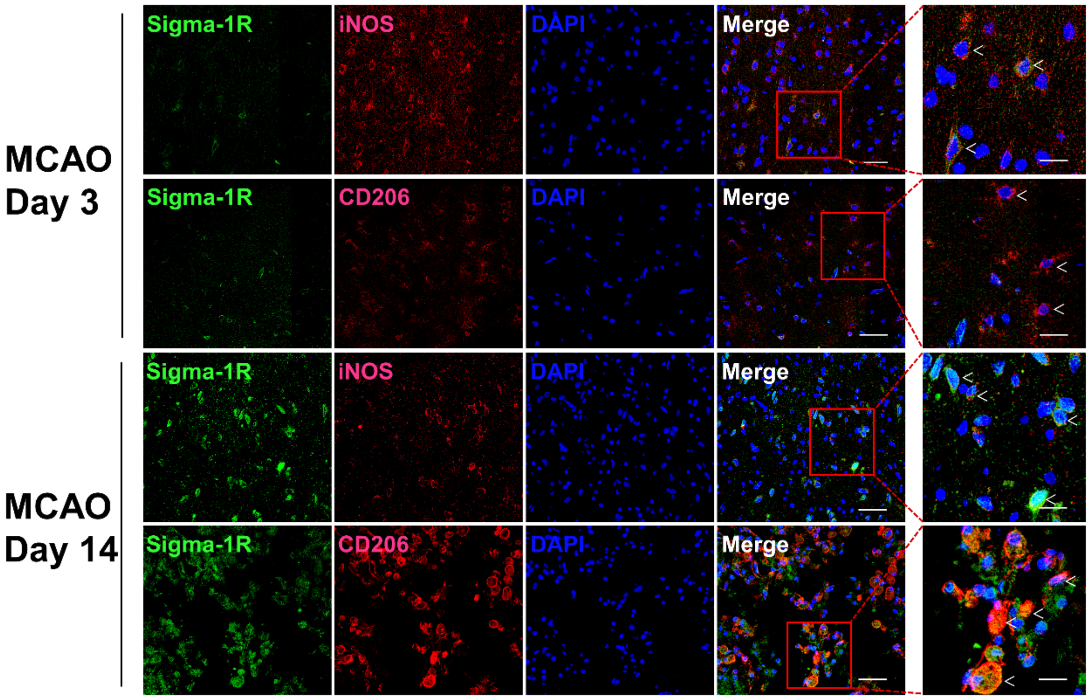

Figure S5. Co-localization of sigma-1R with iNOS or CD206

Immunofluorescence images illustrate the co-localization of sigma-1R (green) with iNOS-positive cells (pro-inflammatory/M1 microglia) or CD206-positive cells (anti-inflammatory/M2 microglia) in the ipsilateral infarcted regions of MCAO rats. White arrows indicate sigma-1R^{+} cells. Scale bars, 50 μm or 20 μm.

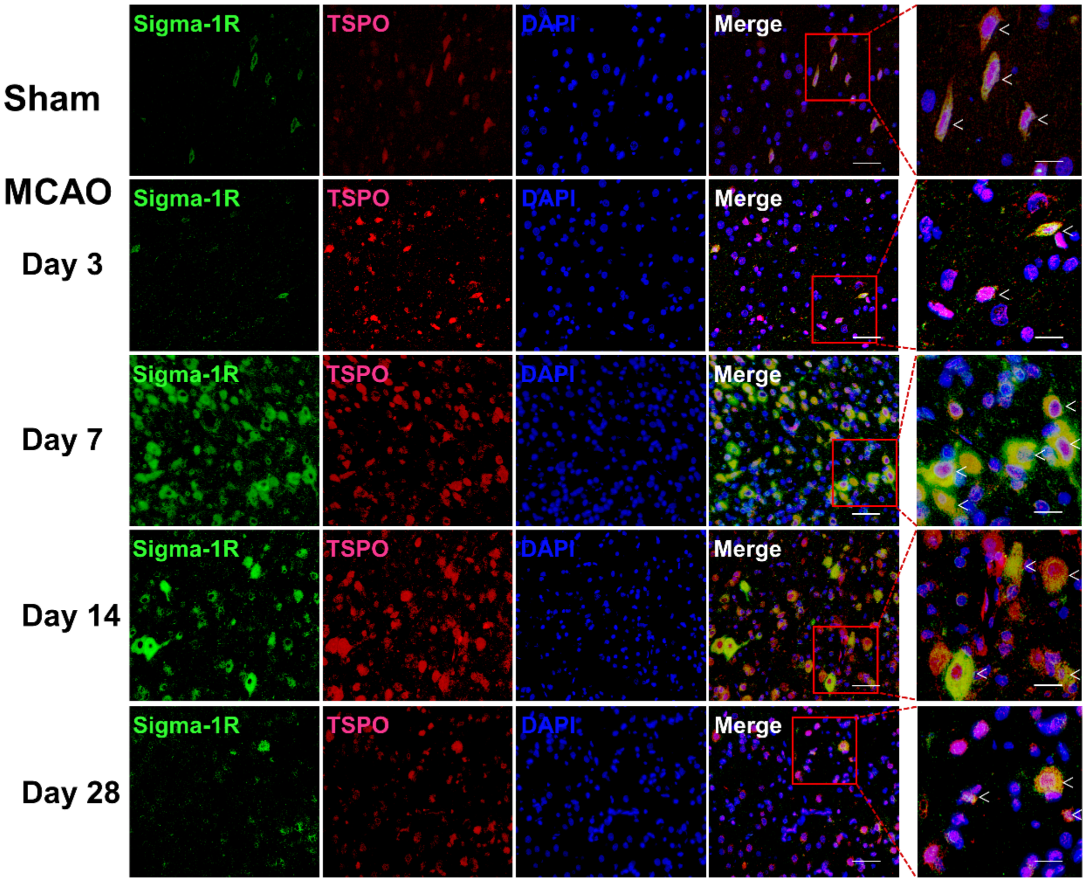

Figure S6. Co-localization of sigma-1R with TSPO

Immunofluorescence images illustrate the co-localization of sigma-1R (green) with TSPO-positive cells (red) in the ipsilateral infarcted regions of MCAO rats on days 3, 7, 14, and 28 post-MCAO. White arrows indicate sigma-1R$^+$ cells. Scale bars, 50 μm or 20 μm.

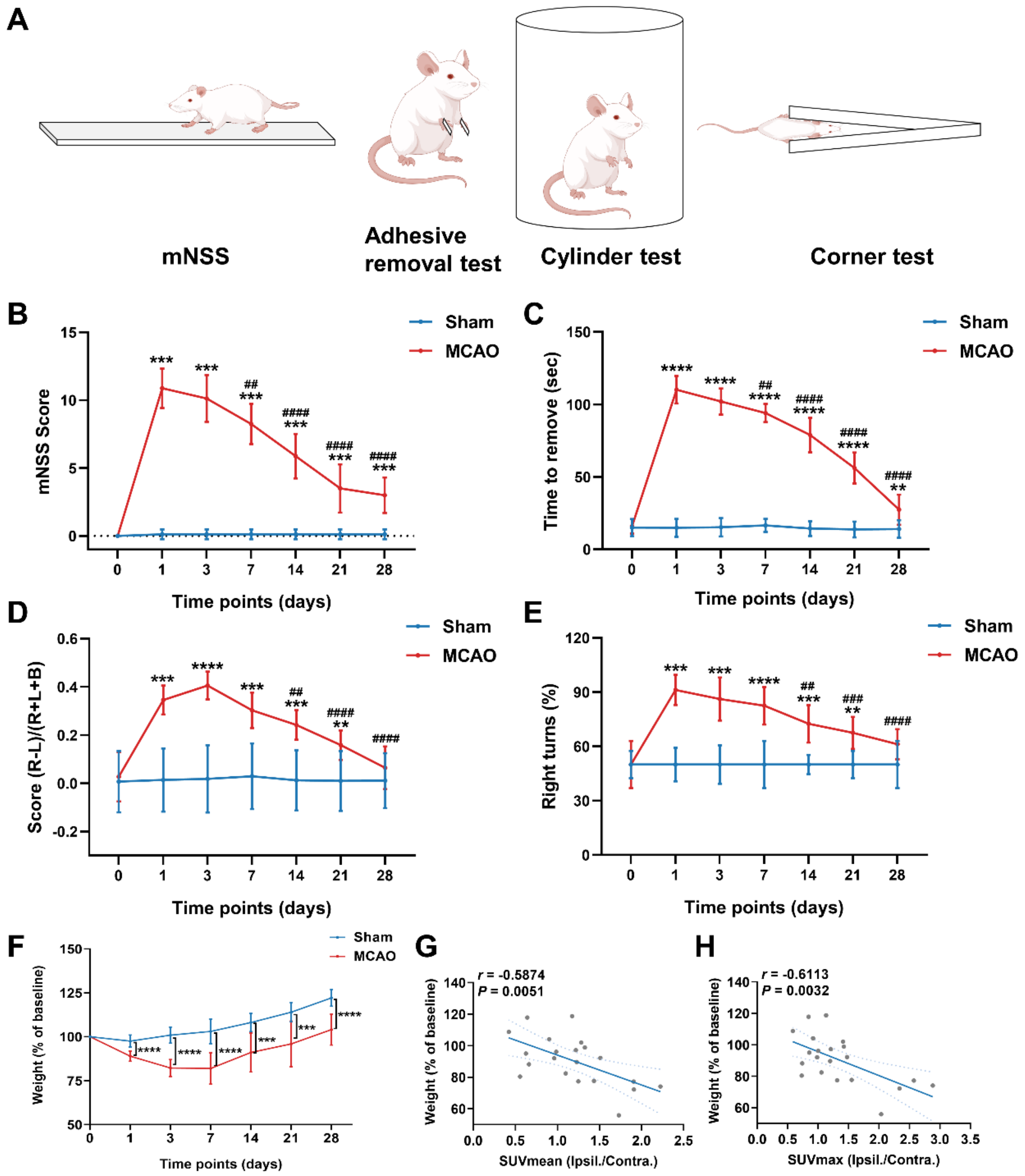

Figure S7. Behavioral results and body weight changes in rats at various time points after ischemic stroke.

(A) Behavioral assessment of sensorimotor function, and schematic diagram was created by Figdraw. (B-E) The mNSS score (B), adhesive removal test (C), cylinder test (D), and corner test (E) were used to evaluate the neurological functions of motor, sensory, and balance after stroke. (F) Changes in body weight in rats from the sham and MCAO groups after stroke. (G, H) Correlation between the ratios of

(R)-[^{18}F]FBFP brain uptake (SUV_{mean} (G) and SUV_{max} (H)) in the ipsilateral hemisphere relative to the contralateral hemisphere and body weight changes in rats from the MCAO group on days 7, 14, and 21 after stroke. Values are mean ± SD (n = 8/group). Statistical significance was calculated with the two-tailed unpaired Student's t-test. Compared to the sham group, $**P < 0.01$, $***P < 0.001$, and $****P < 0.0001$; compared to the MCAO group on day 1 after stroke, $^{\#\#}P < 0.01$, $^{\#\#\#}P < 0.001$, and $^{\#\#\#\#}P < 0.0001$. Correlations were determined using Pearson correlation tests in (G, H). r represents the correlation coefficient and P represents the P value of the correlation test. MCAO, middle cerebral artery occlusion; mNSS, modified neurological severity score; SUV, standardized uptake value.

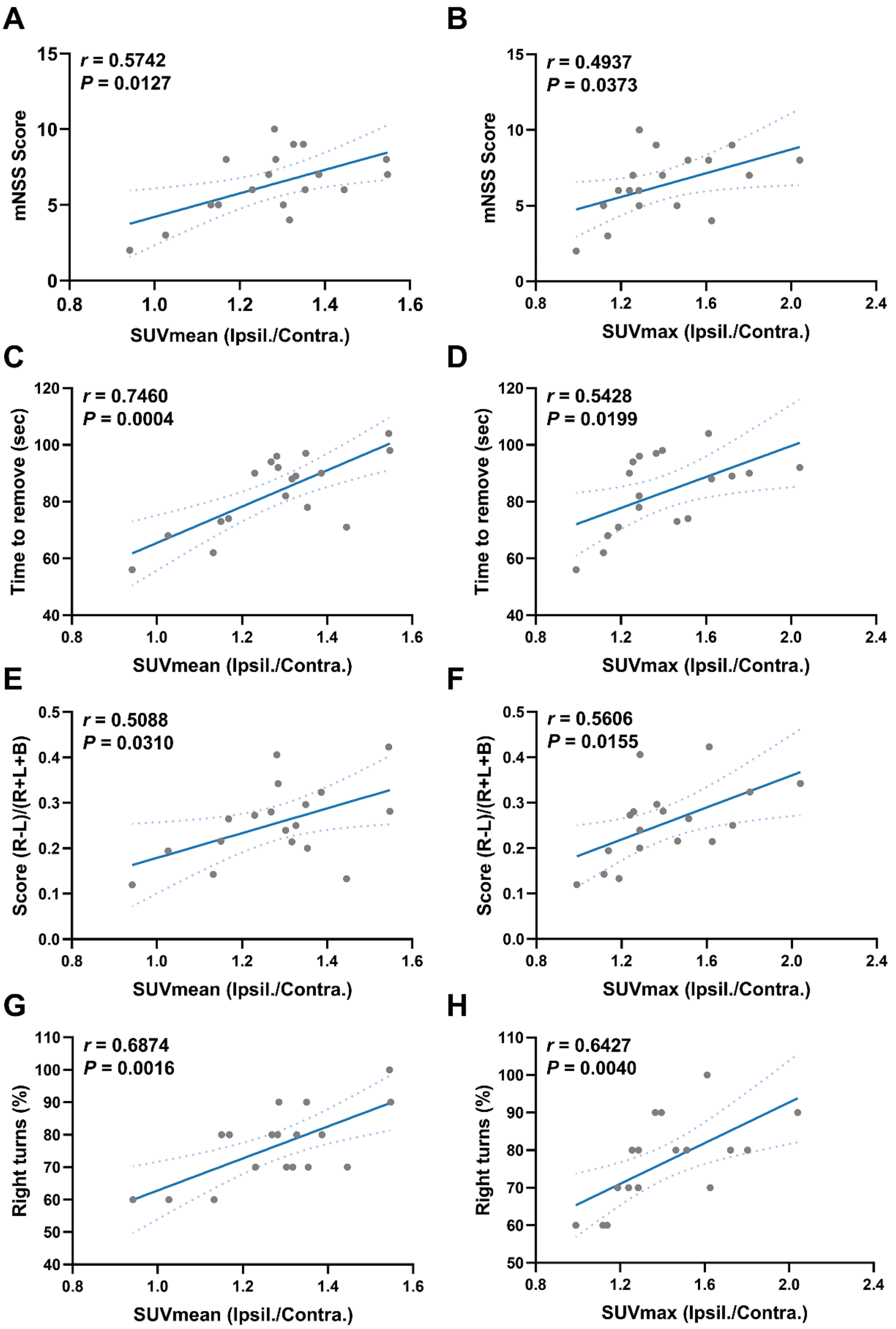

Figure S8. Correlations between *in vivo* (*R*)-[^{18}F]FBFP PET signals and behavioral test outcomes.

(A, B) Correlation between the ratios of (*R*)-[^{18}F]FBFP brain uptake (SUV_{mean} (A) and SUV_{max} (B)) in

the ipsilateral hemisphere relative to the contralateral hemisphere and the mNSS scores in MCAO rats on days 7, 14, and 21 after stroke. (C, D) Correlation between the ratios of (*R*)-[^{18}F]FBFP brain uptake (SUV_{mean} (C) and SUV_{max} (D)) in the ipsilateral hemisphere relative to the contralateral hemisphere and the results of the adhesive removal test in MCAO rats on days 7, 14, and 21 after stroke. (E, F) Correlation between the ratios of (*R*)-[^{18}F]FBFP brain uptake (SUV_{mean} (E) and SUV_{max} (F)) in the ipsilateral hemisphere relative to the contralateral hemisphere and the results of the cylinder test in MCAO rats on days 7, 14, and 21 after stroke. (G, H) Correlation between the ratios of (*R*)-[^{18}F]FBFP brain uptake (SUV_{mean} (G) and SUV_{max} (H)) in the ipsilateral hemisphere relative to the contralateral hemisphere and the result of the corner test in MCAO rats on days 7, 14, and 21 after stroke. Correlations were determined using Pearson correlation analysis. r represents the correlation coefficient and P represents the P value of the correlation test. MCAO, middle cerebral artery occlusion; mNSS, modified neurological severity score; SUV, standardized uptake value.

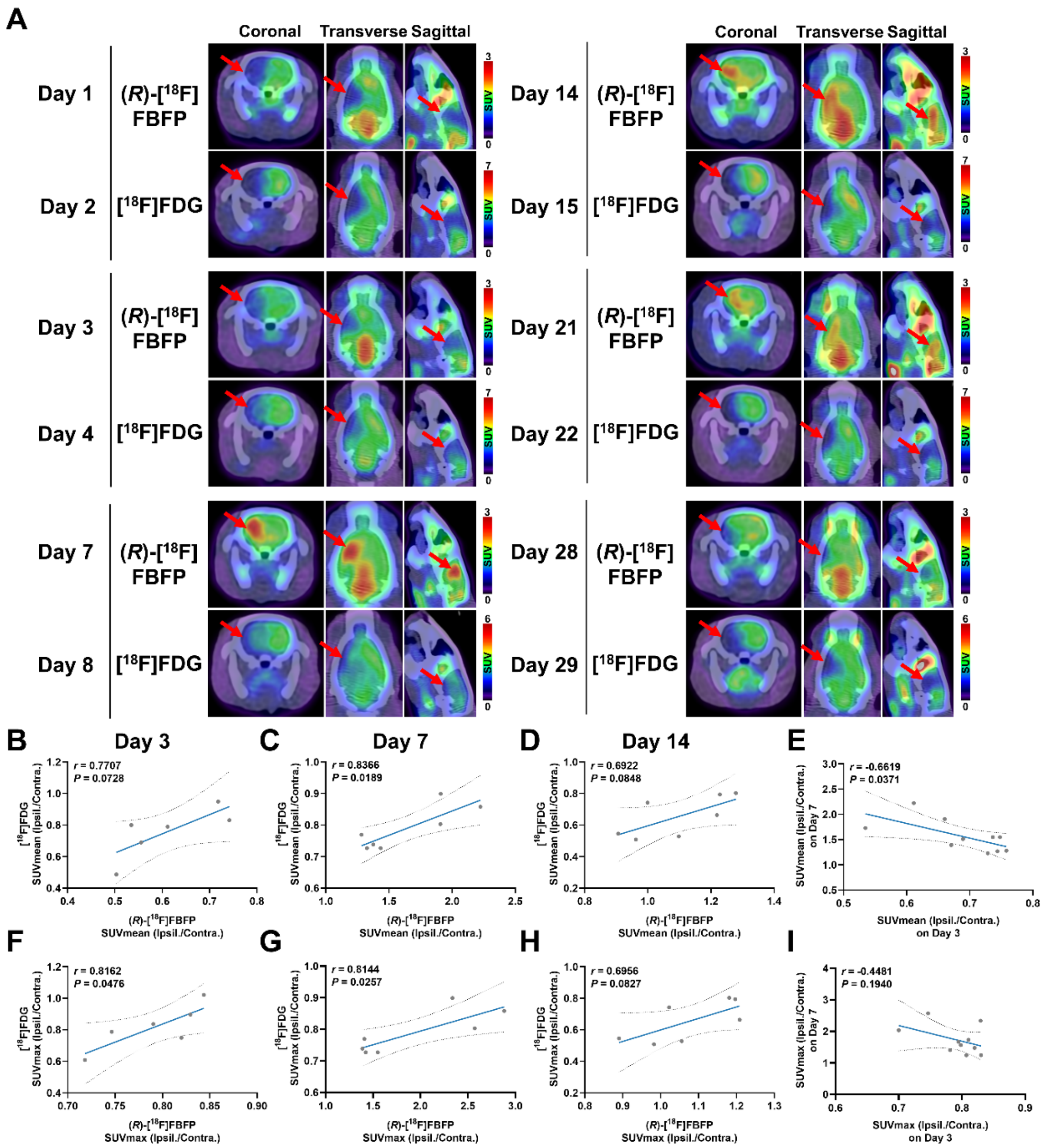

Figure S9. Correlations between (*R*)-[^{18}F]FBFP and [^{18}F]FDG PET signals in MCAO rats

(A) (*R*)-[^{18}F]FBFP PET/CT images of brains from the MCAO rats on days 1, 3, 7, 14, 21, and 28 post-stroke, and [^{18}F]FDG PET/CT images of brains from the MCAO rats on days 2, 4, 8, 15, 22, and 29 post-stroke. Red arrows highlight ischemic lesions in the ipsilateral hemispheres. (B, F) Correlation between the ratios of (*R*)-[^{18}F]FBFP brain uptake in the ipsilateral hemisphere relative to the contralateral hemisphere and the ratios of [^{18}F]FDG brain uptake (SUV_{mean} (B) and SUV_{max} (F)) in MCAO rats on day 3 after stroke. (C, G) Correlation between the ratios of (*R*)-[^{18}F]FBFP brain uptake in the ipsilateral

hemisphere relative to the contralateral hemisphere and the ratios of [^{18}F]FDG brain uptake (SUV_{mean} (C) and SUV_{max} (G)) in MCAO rats on day 7 after stroke. (D, H) Correlation between the ratios of (*R*)-[^{18}F]FBFP brain uptake in the ipsilateral hemisphere relative to the contralateral hemisphere and the ratios of [^{18}F]FDG brain uptake (SUV_{mean} (D) and SUV_{max} (H)) in MCAO rats on day 14 after stroke. (E, I) Correlation between the ratios of (*R*)-[^{18}F]FBFP brain uptake in the ipsilateral hemisphere relative to the contralateral hemisphere on day 3 and the ratios of (*R*)-[^{18}F]FBFP brain uptake (SUV_{mean} (E) and SUV_{max} (I)) on day 7 after stroke. Correlations were determined using Pearson correlation analysis. r represents the correlation coefficient and P represents the P value of the correlation test. MCAO, middle cerebral artery occlusion; SUV, standardized uptake value.

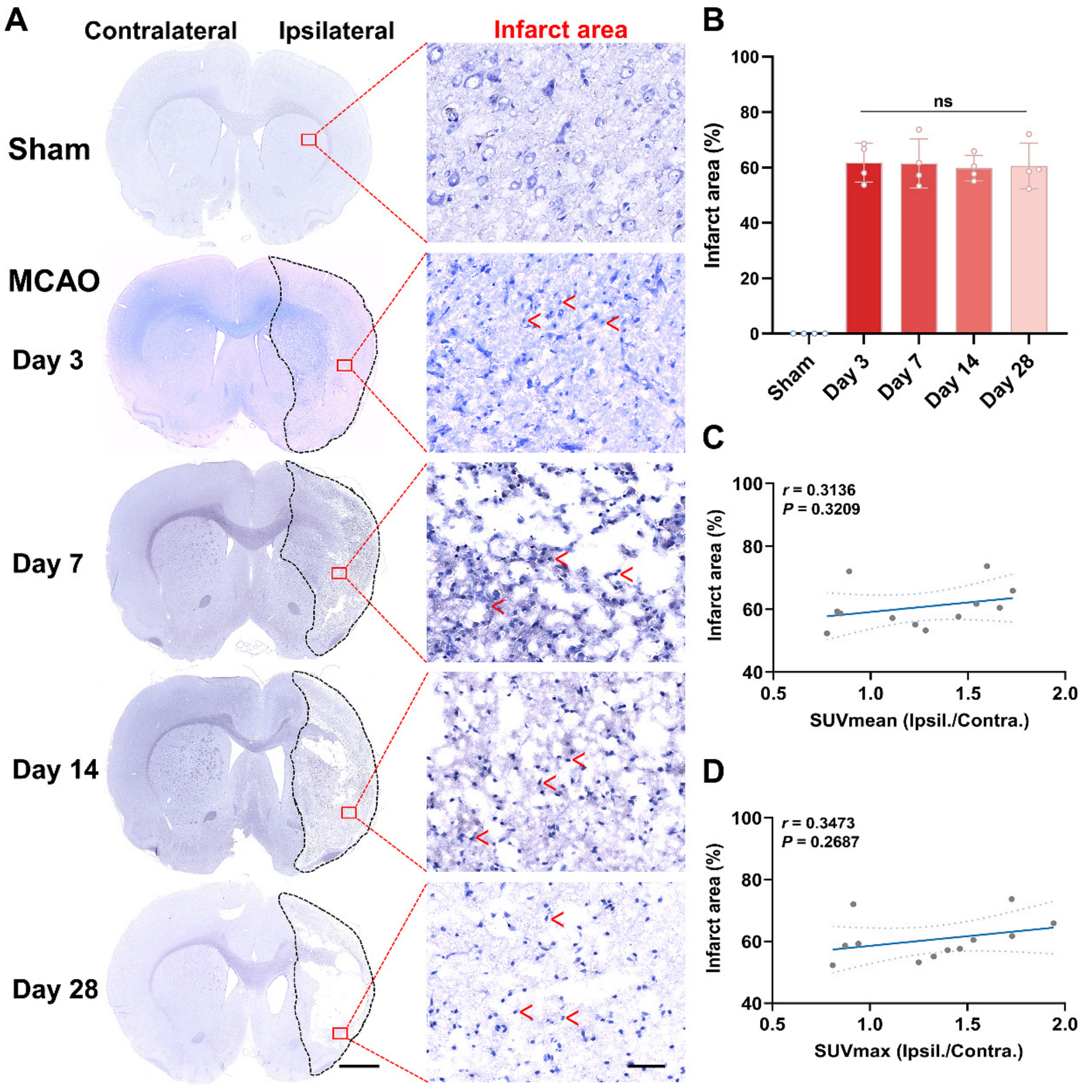

Figure S10. Brain damage in rats after ischemic stroke via Nissl staining.

(A) Representative Nissl staining images of the ipsilateral infarcted regions in rats from the sham group and the MCAO group on days 3, 7, 14, and 28 after stroke. Red arrows indicate the damaged neurons. Scale bars, 2 mm or 50 μm. (B) Quantification of the infarct area based on Nissl staining. (C, D) Correlation between the ratios of (*R*)-[^{18}F]FBFP brain uptake (SUV_{mean} (C) and SUV_{max} (D)) in the ipsilateral hemisphere relative to the contralateral hemisphere and the results of Nissl staining in MCAO rats on days 7, 14, and 28 after stroke. Values are mean ± SD (n = 4/group) in (B). Statistical significance was calculated with the one-way ANOVA test in (B). Compared to the MCAO group on day 3 after stroke,

ns: no significance. Correlations were determined using Spearman's correlation test in (C) and Pearson's correlation test in (D). *r* represents the correlation coefficient and *P* represents the *P* value of the correlation test. MCAO, middle cerebral artery occlusion; SUV, standardized uptake value.

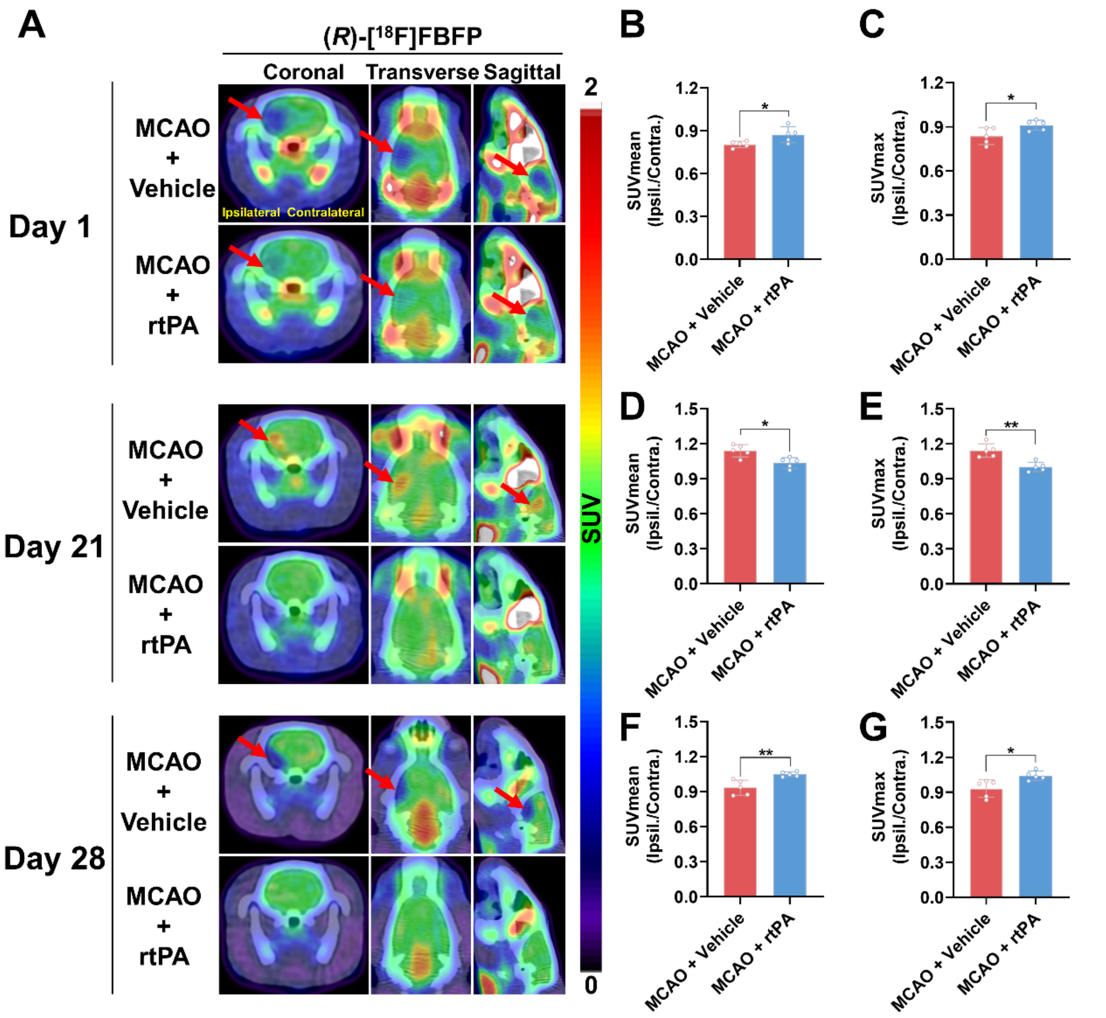

Figure S11. Comparison of (*R*)-[^{18}F]FBFP PET signals between MCAO rats with and without rtPA treatment.

(A) Representative coronal, transverse, and sagittal (*R*)-[^{18}F]FBFP PET/CT images of the brains of rats from the MCAO + Vehicle and MCAO + rtPA groups on days 1, 21, and 28 after stroke, with red arrows highlighting the ischemic lesions in the ipsilateral hemisphere. (B-G) Quantification of the ratios of (*R*)-[^{18}F]FBFP uptake (SUV_{mean} in B, D, F, and SUV_{max} in C, E, G) in the ipsilateral hemisphere relative to

the contralateral hemisphere on days 1, 21, and 28 post-stroke. Values are mean ± SD (n = 5/group). Statistical significance was calculated with the two-tailed unpaired Student's t-test. *$P < 0.05$ and **$P < 0.01$. MCAO, middle cerebral artery occlusion; rtPA, recombinant tissue plasminogen activator; SUV, standardized uptake value.

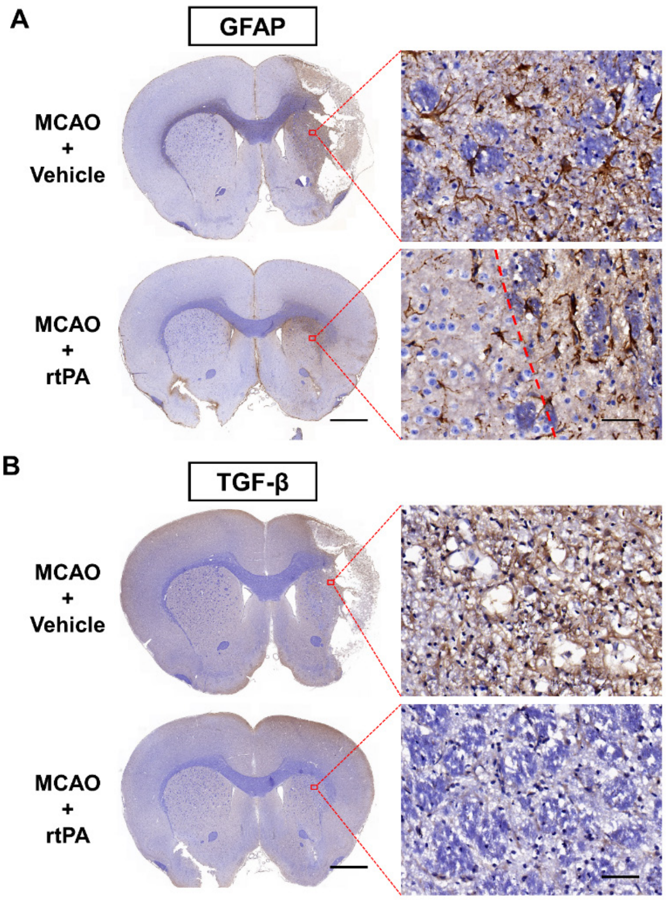

Figure S12. Assessment of rtPA treatment effects on post-ischemic inflammation.

Representative immunohistochemical images for GFAP (A) and TGF-β (B) in MCAO + Vehicle and MCAO + rtPA groups on day 28 after stroke. Scale bars, 2 mm or 50 μm.
